# Thermally Induced Irreversible Disorder in Interlayer Stacking of γ‐GeSe

**DOI:** 10.1002/smll.202407459

**Published:** 2024-10-22

**Authors:** Joonho Kim, Giyeok Lee, Sol Lee, Jinsub Park, Kihyun Lee, Joong‐Eon Jung, Seungjae Lim, Jeongsu Jang, Heesun Bae, Jae‐Ung Lee, Seongil Im, Aloysius Soon, Kwanpyo Kim

**Affiliations:** ^1^ Department of Physics Yonsei University Seoul 03722 South Korea; ^2^ Department of Materials Science & Engineering Yonsei University Seoul 03722 South Korea; ^3^ Center for Nanomedicine Institute for Basic Science (IBS) Seoul 03722 South Korea; ^4^ Department of Physics and Department of Energy Systems Research Ajou University Suwon 16499 South Korea

**Keywords:** γ‐GeSe, atomic resolution transmission electron microscopy, first‐principles calculation, interlayer sliding, Joule heating, stacking disorder

## Abstract

The interlayer stacking shift in van der Waals (vdW) crystals represents an important degree of freedom to control various material properties, including magnetism, ferroelectricity, and electrical properties. On the other hand, the structural phase transitions driven by interlayer sliding in vdW crystals often exhibit thickness‐dependent, sample‐specific behaviors with significant hysteresis, complicating a clear understanding of their intrinsic nature. Here, the stacking configuration of the recently identified vdW crystal, γ‐GeSe, is investigated, and the disordering manipulation of stacking sequence is demonstrated. It is observed that the well‐ordered AB′ stacking configuration in as‐synthesized samples undergoes irreversible disordering upon Joule heating via electrical biasing or thermal treatment, as confirmed by atomic resolution scanning transmission electron microscopy (STEM). Statistical analysis of STEM data reveal the emergence of stacking disorder, with samples subjected to high electrical bias reaching the maximum levels of disorder. The energies of various stacking configurations and energy barriers for interlayer sliding are examined using first‐principles calculation and a parameterized model to elucidate the key structural parameters related to stacking shift. The susceptibility of interlayer stacking to disorder through electrical or thermal treatments should be carefully considered to fully comprehend the structural and electrical properties of vdW crystals.

## Graphical abstract

It is demonstrated that thermal activation induces irreversible disordering in the interlayer stacking of γ‐GeSe, even leading to a fully randomized configuration. The low sliding energy barrier and nearly stacking‐independent energy landscape facilitate this change. These findings highlight the need to consider the susceptibility of interlayer stacking to disorder in various layered crystals.

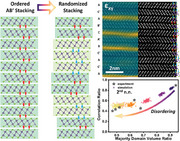

## 1 Introduction

Van der Waals (vdW) layered crystals have garnered significant research attention for their potential applications in nanoelectronics and the exploration of novel phenomena.^[^ ^1^, ^2^, ^3^, ^4^, ^5^, ^6^, ^7^, ^8^ ^]^ Owing to weak interlayer interactions, vdW crystals offer an additional degree of freedom: interlayer stacking configuration. VdW crystals are known to have various polytypes with distinct stacking configurations, which also strongly modulate their physical properties including the band gap of the system.^[^ ^9^, ^10^, ^11^, ^12^, ^13^, ^14^, ^15^ ^]^ In addition, interlayer sliding‐induced ferroelectricity, known as slidetronics, is an emerging research area.^[^ ^16^, ^17^, ^18^, ^19^, ^20^, ^21^, ^22^, ^23^ ^]^ The crystal symmetry of vdW layered materials is tunable by interlayer sliding, which results in the conversion between centrosymmetric and noncentrosymmetric groups as well as the polarization reversal. Recent works show various vdW systems with sliding ferroelectricity including h‐BN, transition metal dichalcogenides (TMDs), 1T′‐ReS_2_, and γ‐InSe.^[^ ^20^, ^21^, ^22^, ^24^, ^25^, ^26^ ^]^ Especially, a ferroelectricity in metallic 1T‐WTe_2_ has attracted significant attention in this field.^[^ ^23^, ^27^, ^28^ ^]^ In general, the polarization switching in these materials is achieved by vertical electric field via local scanning probe microscopy or dual‐gated vertical device geometry.

The disorder in the stacking sequence in vdW layered materials has profound effects in various material properties. The deviation of stacking configuration from a single crystalline configuration is called a stacking fault, and the accumulation of stacking faults results in the disordered stacking configuration in layered crystals. The electrical and thermal transport behavior in disordered stacking has recently acquired significant research interest, especially due to reduced lattice thermal conductivity and improved thermoelectric efficiency.^[^ ^29^, ^30^, ^31^, ^32^, ^33^, ^34^ ^]^ In particular, vdW crystals with disordered stacking, such as turbostratic stacking or heterostructured interfaces in TMDs, exhibit thermal conductivity as low as 0.01 W m^−1^K^−1^ along the out‐of‐layer direction.^[^ ^35^, ^36^, ^37^, ^38^ ^]^ On the other hand, SiC with a lattice constant as large as 150 nm along the c‐axis has been identified, which demonstrates that there are often numerous possible polytypes in the related systems.^[^ ^39^ ^]^ Although the stacking configuration serves as an important way to control the various properties in vdW crystals, the manipulation of interlayer stacking via external stimulus,^[^ ^40^, ^41^, ^42^ ^]^ in particular to disordered stacking configuration, remains a significant challenge.

Here, we investigated stacking sequences in γ‐GeSe using atomic resolution scanning transmission electron microscopy (STEM) imaging in a layer‐by‐layer manner. γ‐GeSe is a recently identified hexagonal polymorph of GeSe with a four‐atom‐thick layer, and its unique local bonding configuration strongly alters its electrical properties compared to archetype GeSe polymorph, α‐GeSe.^[^ ^43^, ^44^ ^]^ In particular, γ‐GeSe displays great potential in thermoelectric and ferroelectric research fields. Recent experimental and theoretical studies show that the γ‐GeSe has low lattice thermal conductivity and spontaneous polarization which can switch its direction by interlayer sliding.^[^ ^45^, ^46^, ^47^, ^48^, ^49^ ^]^ However, there are no experimental studies have been conducted yet on changing its stacking configuration.

In this study, we demonstrated the thermally induced stacking shift disordering in γ‐GeSe devices. The induced stacking disorder can be attributed to the effect of Joule heating under biasing, confirmed by finite element method simulations and observation of similarly disordered stacking sequences in thermally treated samples. The energies of various stacking configurations were calculated using first‐principles calculation and a parameterized model. Our results confirm that the formation energy of the AB′ (AC′) stacking configuration is the lowest, and the energy difference between ordered and disordered stacking is small because the stacking energy is not significantly affected beyond the nearest neighbor (n.n.) layer. Additionally, the calculated energy barrier for interlayer sliding between the AB′ and AC′ stacking configurations is 4.99 meV atom^−1^, consistent with the observed facile formation of stacking disorder generated by the external perturbation. γ‐GeSe with fully disordered stacking can be considered a 1D amorphous system along the c‐axis, offering the potential for adjusting various properties, including thermal conductivity.

## 2 Results and Discussion

**Figure 1:**
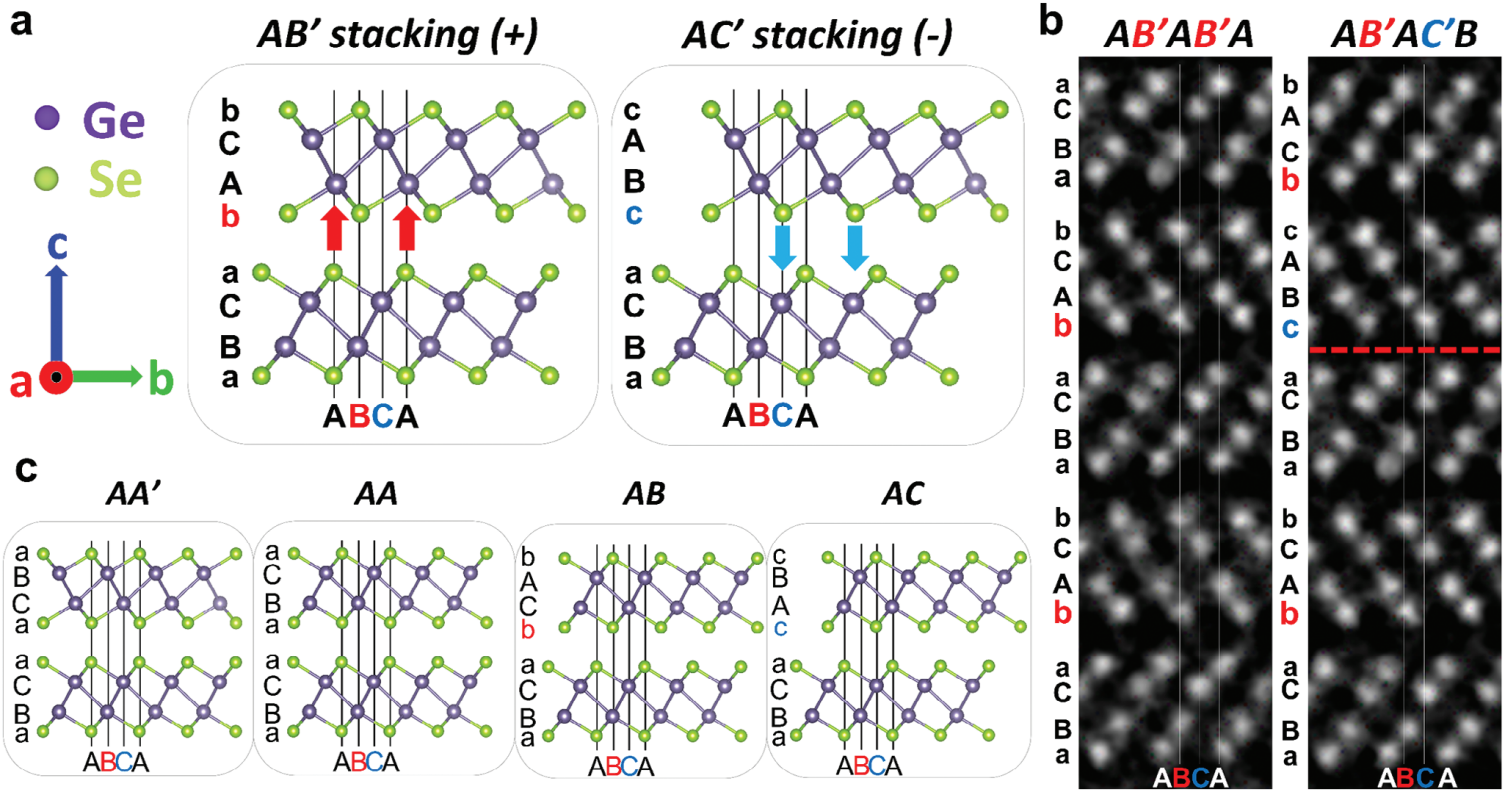
Various stacking configurations in γ‐GeSe. a) Dominant stacking configurations observed in as‐synthesized γ‐GeSe. The vertical arrows indicate the electrical polarization direction from theoretical calculation. b) Cross‐sectional STEM images of γ‐GeSe along the [$10\bar{1}0$] zone axis without and with a stacking fault (red dashed line). c) Other possible stacking configurations in γ‐GeSe which have no electrical polarization.

**Figure** **1** summarizes the possible stacking configurations in γ‐GeSe crystal. Each layer consists of a four‐atom‐thick unit with a sequence of Se‐Ge‐Ge‐Se. The hexagonal lattice of γ‐GeSe has three energetically favorable positions for atomic occupancy, labeled as A, B, and C sites. The sites of top and bottom Se atoms can be denoted as A, and the sites of Ge atoms are denoted as B and C, following the conventional labeling method^[^ ^50^ ^]^ as shown in the bottom layer displayed in Figure 1a. By analyzing the relative positions of γ‐GeSe layers in the exemplary cross‐section STEM images (Figure 1b), we identified two dominant “zig‐zag” stacking sequences (Figure 1a). In an AB′ (AC′) stacking configuration, the upper layer is rotated by 180° about the c‐axis with respect to the lower layer, forming “zig‐zag” stacking sequence. The Se sites between adjacent layers do not occupy the same sites to minimize the stacking energy. AB′ and AC′ have the same structure, which is related by a mirroring with a translation. We designated an AB′ (BC′, CA′) stacking configuration as a “+” and an AC′ (BA′, CB′) stacking configuration as a “−” for convenience. This sign also denotes the direction of electrical polarization as shown by arrows in Figure 1a, as indicated by previous calculation results.^[^ ^48^, ^49^ ^]^ The exemplary STEM images from as‐synthesized samples show the predominant type of zig‐zag stacking (AB′ stacking in the images) under a single‐crystalline stacking configuration (Figure 1b). Another type (AC′ stacking in the image) of zig‐zag stacking serves as a stacking fault, as shown in the right panel of Figure 1b. Figure 1c summarizes the other possible stacking configurations by interlayer sliding, which are absent in as‐synthesized crystals. Owing to the absence of electrical polarization in these stacking configurations, we classified them as “0” configurations in this study.

**Figure 2:**
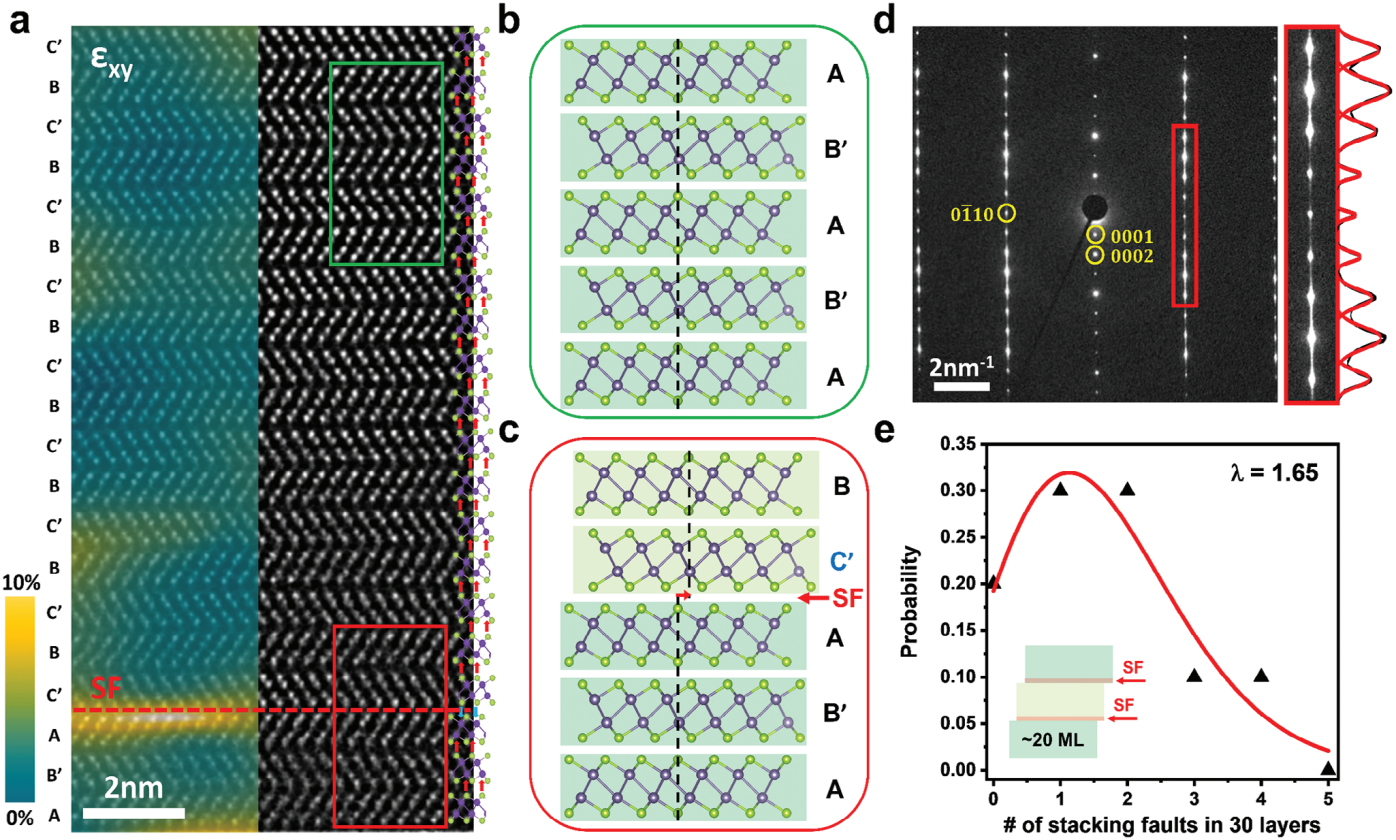
Stacking sequence and domain size analysis of as‐synthesized γ‐GeSe along the $\left[10\bar{1}0\right]$ zone axis. a) Cross‐sectional STEM image of as‐synthesized γ‐GeSe. A stacking fault (SF) is marked with the red dashed line. The strain ε_*xy*_ The value extracted from the STEM image is overlayered on the left side, indicating the presence of an SF. b) Schematic of the stacking sequence observed in the green box indicated in panel a. c) Schematic of the stacking sequence observed in the red box indicated in panel a. d) SAED pattern of as‐grown γ‐GeSe along the [$10\bar{1}0$] zone axis. The curve is the line profile of the SAED intensity. e) The number of observed stacking faults in the unit of 30 layers. The red curve is the fitting to data with a Poisson distribution function. A total of 273 layers from as‐synthesized samples are investigated.

We thoroughly investigated the stacking sequences of the as‐synthesized γ‐GeSe, as shown in **Figure** **2**. The cross‐sectional TEM sample was prepared along the armchair lattice direction of γ‐GeSe using the focused ion beam (FIB) process. From observation along the [$10\bar{1}0$] zone axis, we fully identified the stacking sequences by capturing atomic‐resolution STEM images of γ‐GeSe crystals throughout their thickness (Figure S1a, Supporting Information). Figure 2a shows an exemplary cross‐sectional STEM image of as‐synthesized γ‐GeSe. Our findings indicate that the stacking sequences of γ‐GeSe are well‐ordered with the AB′ stacking configuration across all investigated layers. Nearly all the stacking sequences consist of the “+” configuration, with only a few stacking faults comprising the “−” configuration. The analysis of shear strain ε_*xy*_ by the geometric phase analysis (GPA) with STEM images can easily identify the presence of stacking faults. Notably, there are no “0” configurations in the as‐synthesized γ‐GeSe. Figure 2b,c shows schematic representations observed in the green and red boxes indicated in Figure 2a. The stacking sequence is well‐ordered with AB′ stacking configuration (green box) for most part of the samples, along with a scarce presence of stacking fault associated with the “−” (red box).

We obtained the selected area electron diffraction (SAED) pattern to analyze the presence of the stacking faults over a large sample area (Figure 2d). The SAED pattern confirms the well‐ordered stacking with hints of stacking faults in the as‐grown samples. As shown in the red box of Figure 2d, diffuse lines along the vertical directions can be clearly visualized. These lines originate from the stacking faults in the (0001) basal plane, consistent with the previously discussed STEM image shown in Figure 2a. In contrast, the diffraction signals along the direction of the (0001)‐indexed peak are well‐isolated, as the signal along this direction is solely sensitive to the interlayer distance and not the interlayer stacking configurations. We statistically analyzed the stacking faults of the as‐synthesized γ‐GeSe (Figure 2e), and a histogram of the observed stacking faults in the 30‐layer‐thick analysis unit was prepared. The number of stacking faults in the 30‐layer‐thick unit was well‐fitted by the Poisson distribution function:

(1) $$P\left(n\right)=\frac{\lambda^{n}e^{-\lambda }}{n!}$$

where *n* is the number of stacking faults and *λ* is the mean number of the distribution. The fact that the histogram is well‐fitted by the Poisson distribution indicates that the naturally occurring stacking faults are randomly located in the sample. From the observed mean number *λ* = 1.65, the average domain size of the as‐synthesized γ‐GeSe was estimated to be 20 layers, as depicted in the inset of Figure 2e.

The presence of stacking faults of as‐synthesized γ‐GeSe implies that the interaction across the individual layers is dominated by vdW coupling. The shortest Se–Se distance between adjacent layers is ≈3.76 Å, which is consistent with the doubled vdW radius for Se (3.80 Å).^[^ ^44^ ^]^ Hexagonal Ge_4_Se_3_Te has a crystal structure and stacking configuration similar to γ‐GeSe and is considered to possess a pure vdW gap.^[^ ^51^, ^52^ ^]^ The stacking fault probability of as‐synthesized γ‐GeSe is ≈6% which is far lower than that of Ge_4_Se_3_Te. This difference may originate from the catalytic nature of our synthesis and the absence of atomic disorder in γ‐GeSe.^[^ ^44^, ^53^ ^]^ The pure vdW gap and natural stacking faults in γ‐GeSe show a possibility of controlling the stacking sequence through interlayer sliding.

**Figure 3:**
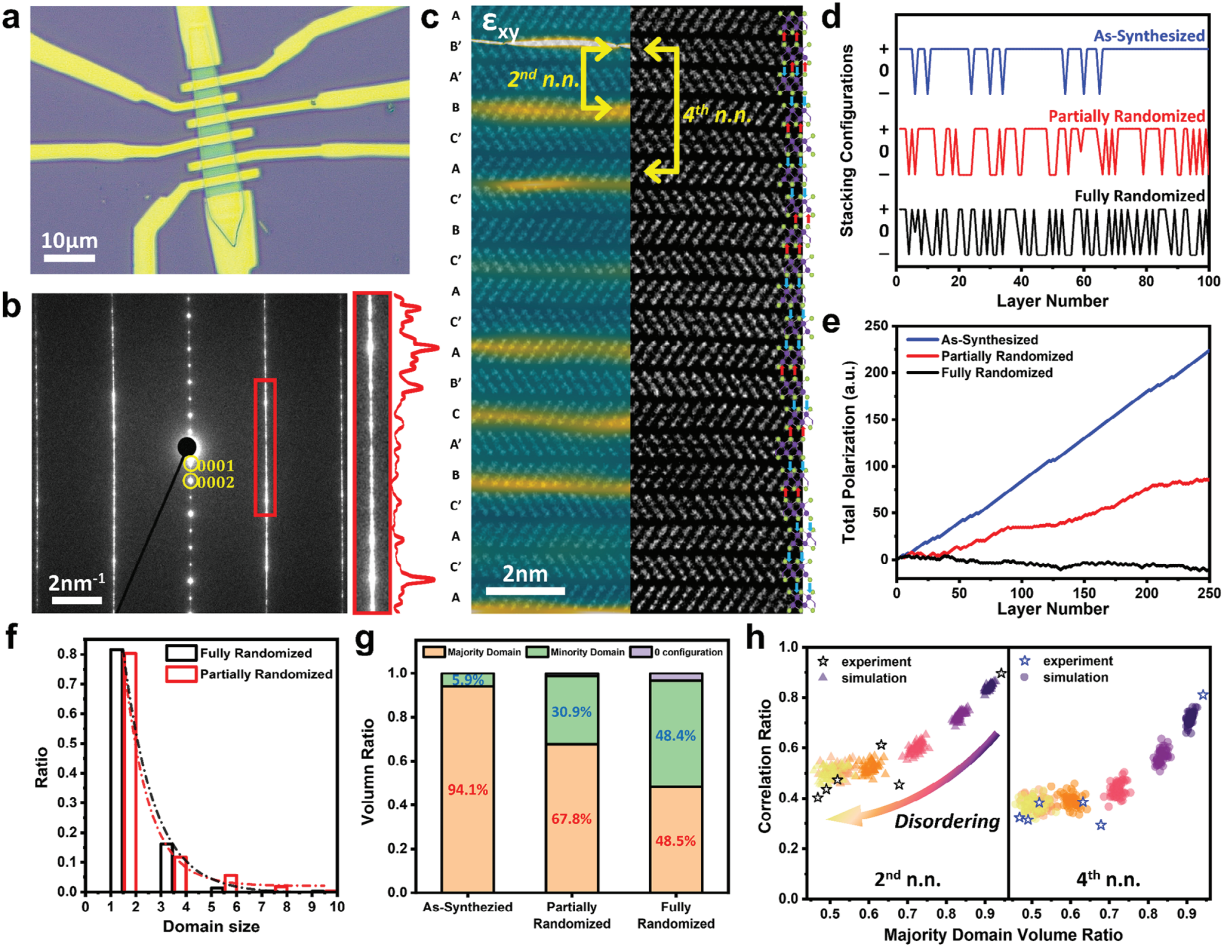
Realization of fully disordered stacking sequence in γ‐GeSe by electric perturbation. a) Optical image of a γ‐GeSe device for electrical perturbation study. b) SAED pattern of electrically‐biased γ‐GeSe along the [$10\bar{1}0$] zone axis. The curve is the line profile of the SAED intensity. c) Cross‐sectional STEM image of γ‐GeSe along the [$10\bar{1}0$] zone axis after electrical biasing. The overlaid strain ε_xy_ color map indicates the formation of a significant number of SFs. d) Sequence of stacking configurations before and after the electrical biasing. e) Comparison of the cumulative electrical polarization before and after the electrical biasing. f) Vertical domain size distributions after the electrical biasing. The dotted curves are the fitting to data with an exponential decaying function. A total of 2005 layers from fully randomized samples and 978 layers from partially randomized samples are investigated. g) Ratio between different stacking domains before and after the electrical biasing. h) Comparison of the 2nd and 4th n.n. correlation between experimental observation and Monte Carlo simulations.

We investigated the active manipulation of stacking configurations of γ‐GeSe under electrical biasing. For this purpose, we fabricated electrical devices using standard e‐beam lithography (**Figure** **3**a). The example of electrical biasing using two‐terminal device geometry is shown in Figure S2 (Supporting Information). Owing to the high level of *p*‐doping, the γ‐GeSe exhibited high conductivity exceeding 5 × 10^5^ S m^−1^.^[^ ^43^ ^]^ The *I–V* characteristics exhibited a linear curve at low operating voltage (0.1 V) without any hysteresis (Figure S2a, Supporting Information). Under relatively high voltage, the device remained in ohmic behavior (Figure S2b, Supporting Information). However, we observed a permanent increase in resistance, suggesting a structural alteration in the crystal. After applying a relatively strong bias, we measured the *I–V* curve at low voltage again and confirmed that the resistance of the device had increased by 4.5% (Figure S2a, Supporting Information). After electrical biasing, we observed a significantly different interlayer stacking sequence from cross‐section STEM imaging and electron diffraction, as shown in Figure 3b,c and Figure S1b (Supporting Information). The analysis confirms that γ‐GeSe samples with increased resistance exhibited a significant increase in stacking faults compared to as‐grown γ‐GeSe. We also observed the presence of “0” configurations after electrical biasing. The strain analysis by GPA clearly visualized the numerous formations of different stacking domains as shown in Figure 3c. The increased diffuse lines in the SAED pattern also confirmed the increased stacking disorder throughout the whole sample (Figure 3b).

We performed the statistical analysis using cross‐sectional STEM images with 3000 layers from multiple FIB‐processed device samples. The sequence of stacking configurations can be assigned as “+”, “−”, and “0” depending on the expected polarization as shown in Figure 3d. The one type of configuration (here, “+” configuration) dominates in as‐synthesized samples with few stacking faults, as explained previously. The disordering of the stacking configuration after the electrical biasing can be visualized by this analysis. Figure 3e shows the cumulative sum, or total electrical polarization, of the data in Figure 3d, where we assigned values of 1, 0, and −1 to “+”, “0”, and “−” configurations, respectively. The slopes of the three categories are clearly distinct. As‐synthesized samples display the highest slope of 0.91. On the other hand, the data shown in black converges to 0, indicating that the sample is maximally randomized samples. Figure 3f presents a statistical analysis of the vertical domain size from disordered samples, in which the distribution is well‐fitted with the exponentially decaying function. We also examined the in‐plane correlation of the stacking sequence between different local regions (Figure S3a, Supporting Information). Figure S3b (Supporting Information) presents exemplary STEM data used for the in‐plane stacking correlation analysis, obtained from the orange and red boxes in Figure S3a (Supporting Information). Our findings revealed that the stacking sequences remained largely identical, with over 95% correlation, even across distances of several micrometers (Figure S3c, Supporting Information). This data confirms that the layer shift is mostly continuous throughout the samples, with occasional discontinuities likely caused by defects, such as partial dislocations in the layer shift. We also analyzed the proportion of different stacking configuration domains (Figure 3g). The portion of dominant stacking domains decreased from disordering, and the portion of “+” and “−” configurations were almost identical at 48.5% and 48.6% respectively in fully randomized samples.

We conducted Monte Carlo simulations of stacking disordering and compared key parameters with experimental results to elucidate the disordering. Starting from 499 well‐ordered “+” configuration (500 layers) sets, we randomly selected one stacking configuration and flipped it to the opposite configuration (“+” to “−” and vice versa) to simulate the process of interlayer sliding. We generated six different 500‐layer sets, where stacking flips were applied 50, 100, 200, 400, 800, and 1600 times respectively. We then extracted nearest neighbor (n.n.) stacking correlations and a portion of “+” domain from resulting disordered sets. The n.n. correlation values were defined as the probability of observing the expected stacking configuration from an ideal well‐ordered single‐crystalline sample.

Figure 3h displays 2nd (and 4th) n.n. correlation along with the portion of “+” domain in samples. Results for 2nd n.n. were presented in a left panel and those for 4th n.n. were displayed in a right panel. As the number of stacking flips increased (shown in different colors from violet to yellow), the portion of the “+” domain decreased (approaching 50%), and n.n. correlations converged to specific values (50% for 2nd n.n. and 37.5% for 4th n.n., respectively). After 1600 stacking flips (shown in yellow triangles), the samples reach a state of maximum disorder. The experimental results follow similar trends observed in the simulation data. The n.n. stacking correlation values from the as‐synthesized γ‐GeSe was significantly high (89.6% for the 2nd n.n. and 81.1% for the 4th n.n.). γ‐GeSe samples influenced by electrical bias exhibited substantially lower correlation values. Fully randomized samples have a majority domain ratio of ≈50%, with average 2nd and 4th n.n. correlations being 42.7% and 33.8%, respectively. These results indicate that the stacking sequences of these samples were randomized to the maximum extent through interlayer sliding. The slight deviation between the experiment and simulation originated from the “0” configuration, which is not considered in our simulations. The observed fully randomized stacking can be considered an 1D amorphous state along the c‐axis which is unique in layered materials.

**Figure 4:**
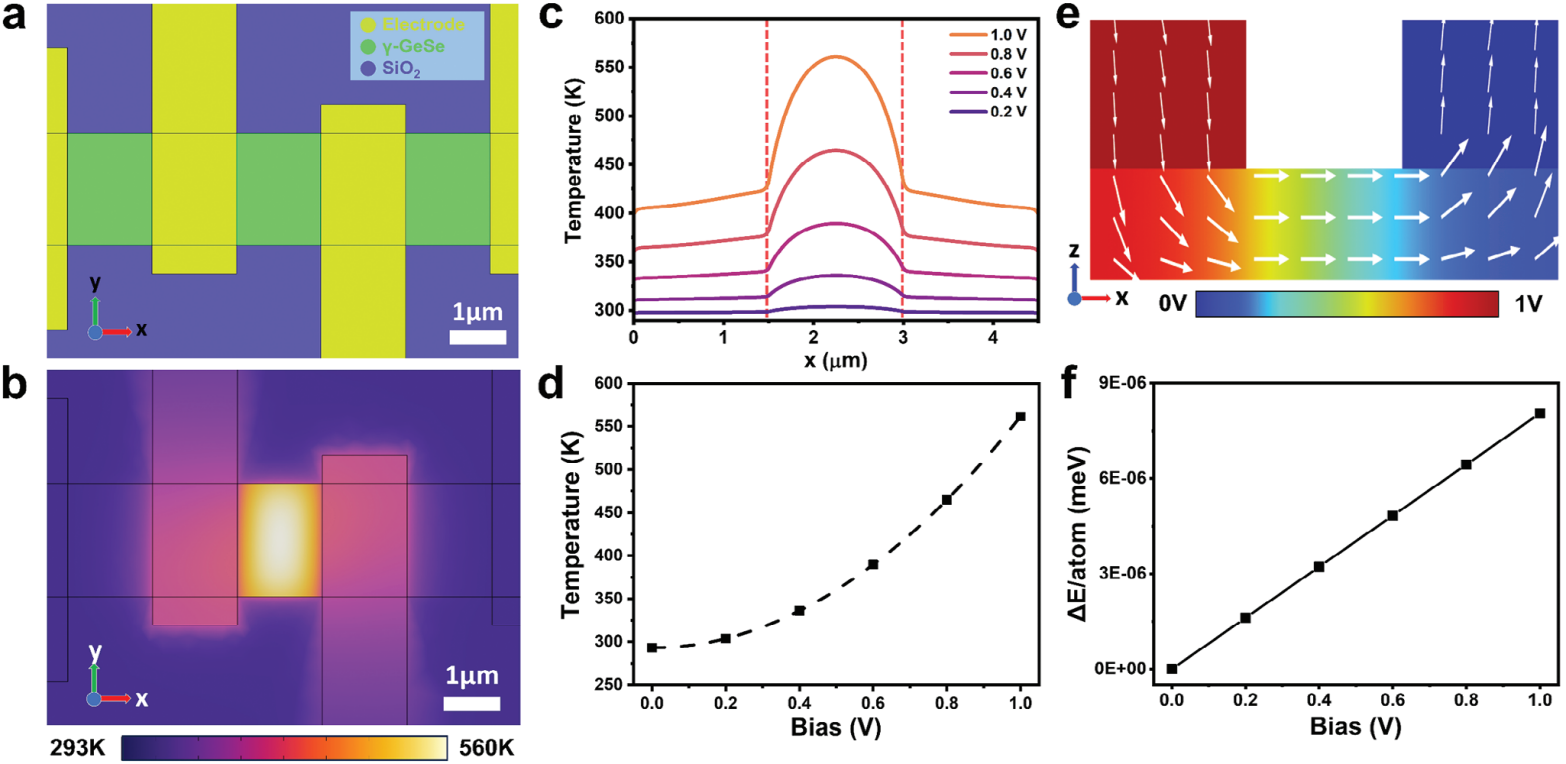
FEM simulation of a γ‐GeSe device under electrical biasing. a) Top‐view schematic of a γ‐GeSe device for simulation. b) Temperature distribution of the device under electrical biasing of 1.0 V. c) Temperature line profiles across the center region of the device at various biasing voltages. d) Local maximum temperature of γ‐GeSe under electrical biasing. e) Cross‐sectional electric potential map in γ‐GeSe device under electrical biasing of 1.0 V. White arrows represent the strength and direction of the local electric field. f) Maximum potential energy difference of the electric dipole of γ‐GeSe in an electric field.

To understand the mechanism of stacking disordering observed in γ‐GeSe devices, we performed finite element method (FEM) simulations to estimate Joule heating and the electric field effect during biasing. The device geometry identical to the electrical biasing experiments was utilized in the simulations as shown in **Figure** **4**a and S5a (Supporting Information). We referenced the electrical and thermal conductivity of γ‐GeSe from our previous results^[^ ^43^, ^45^ ^]^ to simulate the temperature increase by the Joule heating. The key parameters also include the electrical contact resistance between the electrodes and γ‐GeSe, as well as the thermal boundary conductance between γ‐GeSe and the SiO_2_/Si substrate.^[^ ^54^ ^]^ Detailed considerations of the FEM simulation equation and parameters are provided in Supporting Note and Figure S4 (Supporting Information).

FEM simulation results show that there is significant Joule heating from our device operation condition (1.0 V biasing) as shown in Figure 4b. The region under the electrode experiences relatively low temperatures due to effective heat dissipation, while the central region of the channel exhibits the highest temperature, making a temperature gradient across the sample. Figure 4c represents a line profile of the temperature map in the x‐direction from Figure 4b. As the bias increases, the temperature of γ‐GeSe rises by several hundred degrees, indicating a significant Joule heating effect which is enough to overcome the energy barrier associated with interlayer sliding. The temperature increases quadratically depending on the bias voltage as shown in Figure 4d. Figure S5b (Supporting Information) is the exemplary temperature estimation of partially randomized and fully randomized samples. The sample exposed to a maximum of 1.2 V reached 332 K and became partially randomized, whereas the sample exposed to a maximum of 1.7 V reached 523 K and became fully randomized. The different slope of the two curves is due to the differences in contact resistance and geometry of the two samples.

In addition, we calculated the electric field effect in the device under bias. The color map of Figure 4e is the electric potential and the white arrows represent the magnitude and direction of the electric field within the sample. The horizontal component of the electric field dominates since the channel length is much larger than the thickness of γ‐GeSe (Figure S5c,d, Supporting Information). Figure S5e,f (Supporting Information) is the line profile of the electric field in the x‐direction from Figure S5c,d (Supporting Information) which also shows the dominance of the horizontal field. We roughly calculated the maximum potential energy difference of electric dipole in γ‐GeSe using a vertical electric field which could switch the direction of polarization (Δ*E*  =  2*p* · *E_z_*).^[^ ^48^ ^]^ The calculated energy is significantly smaller than the energy barrier for interlayer sliding which will be discussed later (Figure 4f). Therefore, we conclude that the stacking disordering in γ‐GeSe is mainly induced by the Joule heating effect rather than the electric field effect.

The investigation of stacking configurations from thermally annealed samples was performed to complement our analysis. To prevent the evaporation of γ‐GeSe, we deposited a 50nm‐thick of Au on the sample and annealed it to 623 K for 1.5 h in an N_2_ atmosphere, and then prepared a cross‐sectional TEM sample using the FIB process (Figure S6a,b, Supporting Information). We heated the sample to a sufficiently high temperature to clearly observe the disordering effect. The presence of substantial stacking disorder throughout the sample was evident in diffuse lines of the SAED pattern, which was further confirmed by the STEM image (Figure S6c,d, Supporting Information). The distribution of domain size is well‐fitted with the exponentially decaying function, revealing a high proportion of small‐sized domains and indicating a considerable degree of disordering (Figure S6e, Supporting Information). Therefore, we confirmed that thermal annealing also can induce stacking disorder in samples (Figure S6f, Supporting Information). The second harmonic generation (SHG) is often utilized to study the crystal symmetry and stacking configurations in layered materials.^[^ ^55^, ^56^, ^57^, ^58^ ^]^ We observed that the SHG signal from γ‐GeSe decreased after the thermally annealing at 623 K as shown in Figure S6g (Supporting Information). The randomized stacking configuration leads to decreased net electrical polarization, and the reduced SHG is consistent with our expectation.

To understand the interlayer stacking configurations in γ‐GeSe, the interlayer stacking energies were investigated using first‐principles calculations and related analysis. Because of possible various stacking configurations in γ‐GeSe, we adopted the theoretical framework from a recent work, which investigated the relative thermodynamic stabilities in post‐transition metal chalcogenides.^[^ ^50^ ^]^ Compared to these post‐transition metal chalcogenides in this study, γ‐GeSe exhibits a unique intralayer atomic sequence, which warrants a detailed comparison study. The employed theoretical linear models parameterized the stacking energies with important stacking features. First, we considered all possible stacking configurations with the following two assumptions: i) Ge and Se atoms are positioned at the A, B, and C hexagonal sublattice sites, based on the strong likelihood of energy minima occurring at these high‐symmetry sites. ii) The hexagonal sublattice sites of the successive Se‐Ge‐Ge‐Se quadruple atomic units maintain a clockwise (A‐B‐C‐A) or counterclockwise (A‐C‐B‐A) configuration to preserve the structural identity of monolayer (ML) γ‐GeSe. As shown in Figure S7 (Supporting Information), a revised version of the stacking sequence redundancy review algorithm was employed to remove the overlapping identical stacking configurations.^[^ ^50^ ^]^ This allowed us to consider all but unique stacking sequences as summarized in Table S1 (Supporting Information). Finally, to reveal the local geometric features of a stable stacking sequence using the generated structures, we constructed a linear equation based on 4 geometric features from the nearest neighboring monolayer and 7 from the next‐nearest neighboring monolayer. For a schematic of these descriptions, please refer to Figure S8 (Supporting Information).

Using this approach, the energy of each structure *P* for a specific stacking sequence can be expressed by the following linear equation:

(2) $$E\left(P\right)=E_{c}+\frac{1}{N_{1}\left(P\right)}\left[\sum n_{k}\left(P\right)E_{k}+\sum n_{l}\left(P\right)E_{l}\right]$$

where *N*_1_(*P*) is the number of monolayers in the structure *P*, and *E_C_* is the normalized total energy per monolayer unit cell of the AA superlattice, *n_k_* is the number of geometric features from the nearest neighboring monolayer, *n_l_* is the number of geometric features from the next‐nearest neighboring monolayer, and *E_k_* and *E_l_* are the coefficients of each geometric feature, determined by fitting the linear equation.

Here, the energies of all distinctive stacking sequences up to 4 monolayers were fitted into the linear equation after conducting first‐principles calculations. To avoid highly correlated parameters that significantly reduce the interpretability of the regression, we measured the variance inflation factor (VIF) for each parameter in all combinations of 11 geometric features (a total of 2035 cases). We then selected the combination with the highest *R* ^2^ value while ensuring all parameters had VIF values <3, resulting in a linear equation comprising 8 parameters. Details are provided in the Supporting Note.

**Figure 5:**
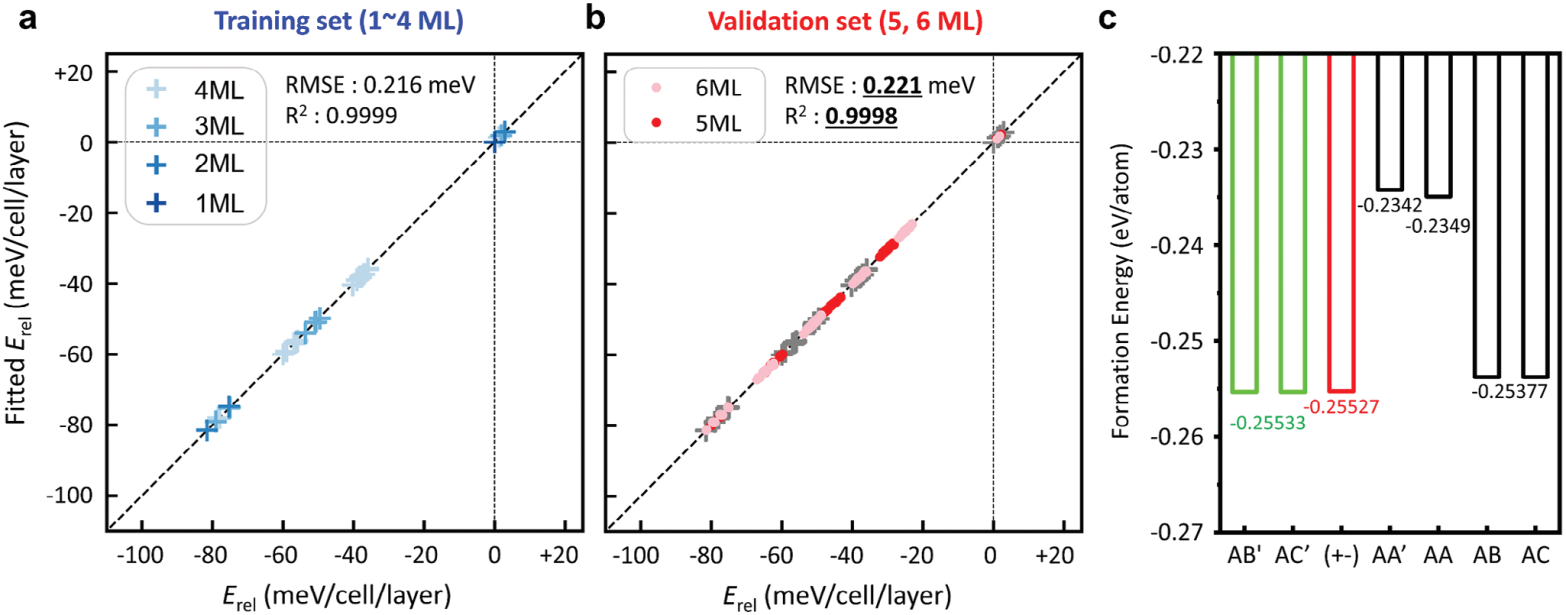
Comparison of predicted results (Fitted *E*_rel_) versus DFT results (*E*_rel_). 259 unique structures up to 5 ML are optimized along the z‐axis, and used for linear regression of Equation. (2), using the least‐squares method. For 6 ML structures, 438 are randomly chosen for validation. a) Generated structures until 4 ML are used as training dataset b) 5 and 6 ML structures are used for validating the fitted equations. c) Formation energies of various interlayer stacking configurations in γ‐GeSe.

Notably, the linear equation used in this study can be extended to stacking sequences for even higher multi‐layer superlattices, surpassing 4 monolayers. As shown in **Figure** **5**b, this was validated by comparing the total energies obtained from first‐principles calculations with those predicted by the linear equation for superlattices with 5 and 6 monolayers, resulting in a negligible RMSE of 0.22 meV/cell/layer.

From our density functional theory (DFT)‐parameterized model, for two neighboring monolayers, it is thermodynamically favorable to maximize the number of neighboring Se atoms on different hexagonal sublattice sites and the number of quadruple atomic units of neighboring monolayers that have different hexagonal sublattice configurations. This implies that the nearest neighboring monolayers have a local stacking sequence of AB' (AC') superlattice (Figure 5c). When extended to the next‐nearest neighboring monolayers, as shown in Figure S9 (Supporting Information), a slight energy increase is observed when the polarization direction changes. This indicates that the stacking sequence with an aligned polarization direction in as‐synthesized samples is indeed more thermodynamically stable. However, even when the polarization direction changes, the energy increase is very minute, with fully disordered configurations showing an increase of 0.24 meV per cell per layer. These results are consistent with the observed facile formation of fully disordered stacking configurations in γ‐GeSe.

**Figure 6:**
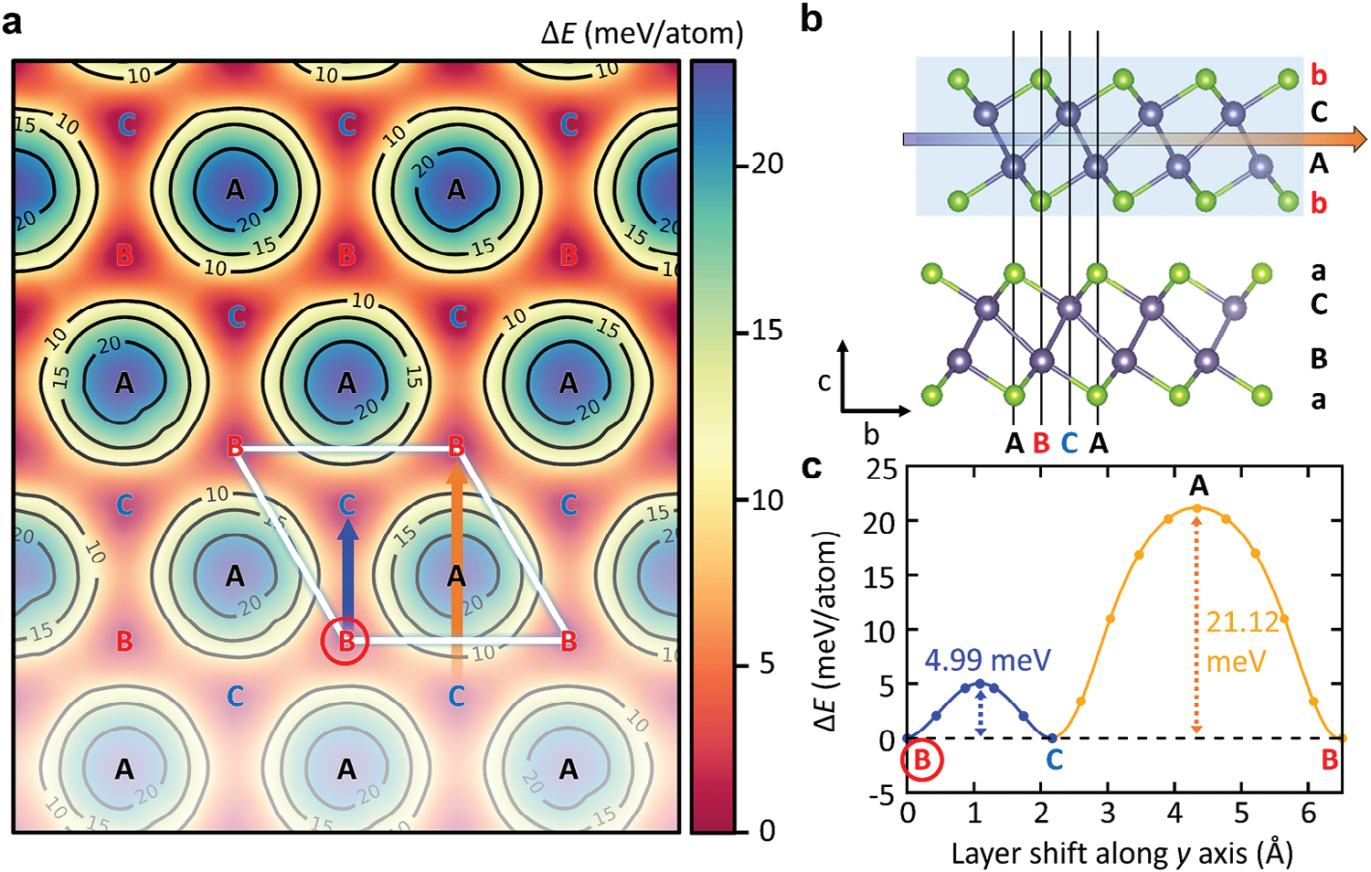
The energy barrier for interlayer sliding in γ‐GeSe. a) Top view of potential energy surface under the shifting of the upper γ‐GeSe layer starting from AB′ stacking configuration. The reference value for the energy is from the AB′ stacking, where the location of lower Se in top monolayer γ‐GeSe is positioned at B hexagonal sublattice. b) Side‐view schematics of AB′ stacked γ‐GeSe with interlayer sliding. c) Energy profile along the arrows in panel a.

Another aspect of the fully disordered stacking that requires verification is the energy barrier associated with the interlayer sliding between two adjacent monolayers. To investigate this phenomenon, we conducted first‐principles calculations based on the most stable two‐layer AB' γ‐GeSe, as illustrated in **Figure** **6**. The energy difference Δ*E* of displacing the upper layer with respect to the lower layer is shown in Figure 6a. For a more quantitative analysis, the Δ*E* of interlayer sliding is depicted in Figure 6c. The energy barrier of interlayer sliding along the blue arrow (B→C) is approximately 4.99 meV atom^−1^, which is approximately four times lower than that along the orange arrow (C→A→B). Notably, when the upper layer slides along the direction of the blue arrow, it becomes AC', which is identical to AB' flipped along the a‐axis. Therefore, when applying electrical perturbation, interlayer sliding can easily occur and stabilize at the AB' or AC' configuration, which has the lowest energy. Furthermore, the calculated energy barrier for the interlayer sliding is comparable to other layered materials,^[^ ^59^, ^60^, ^61^, ^62^, ^63^ ^]^ making it easily overcome by mild external control, such as ambient temperature and Joule heating (Table S2, Supporting Information).

**Figure 7:**
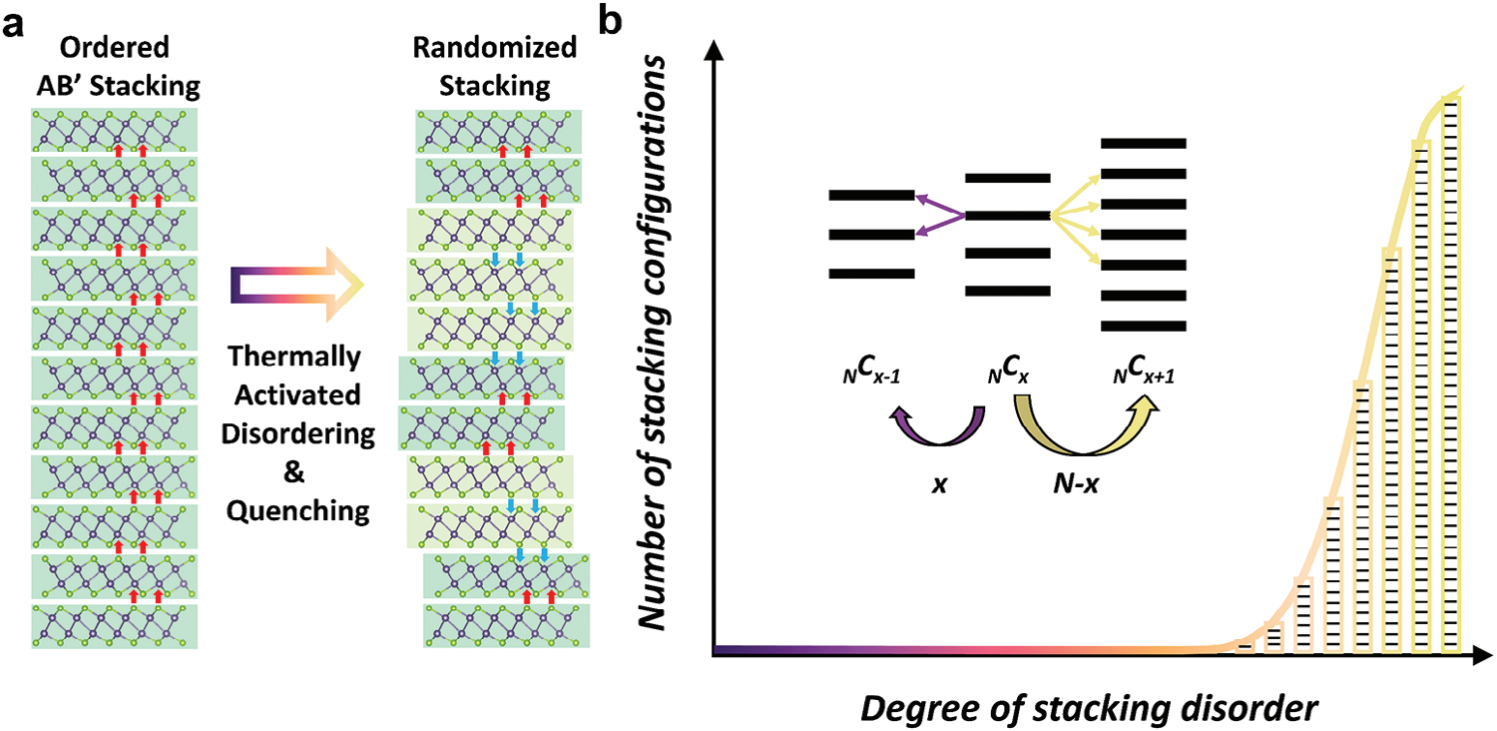
Schematic summary of the stacking disordering observed in γ‐GeSe. a) Schematic of the stacking disordering process observed in γ‐GeSe. b) The number of energetically degenerate stacking configurations which drastically increases with the level of disorder.

**Figure** **7** shows the schematics of the observed stacking disordering in γ‐GeSe. The as‐synthesized γ‐GeSe crystals with well‐ordered stacking configurations can be disordered by Joule‐heating or thermal annealing processes. The energy barrier for interlayer sliding is quite small and the sliding can be activated at elevated temperatures. On the other hand, the energy difference between different stacking configurations is marginal, and these configurations can be considered as energetically degenerate. If we consider the possible number of stacking configurations as a function of the level of disorder, the majority of configurations is dominant near the maximally disordered state as shown in Figure 7b. The interlayer sliding promoted at elevated temperature induces the transition between these energetically degenerate configuration spaces and the final outcome will be the randomized stacking configurations. Once cooling, the samples can be quenched into disordered stacking configurations, resembling the amorphous state of matter.

## 3 Conclusion

In this study, we investigated the stacking disordering of the newly emerging layered crystal, γ‐GeSe, using atomic resolution STEM imaging and theoretical calculations. We adopted in‐plane device geometry to achieve randomized stacking, in contrast to the general vertical geometry used for ordering the stacking sequence. Well‐ordered AB′ stacking configurations in the as‐synthesized γ‐GeSe were randomized via the Joule heating effect. Controlling the stacking configuration is crucial for tuning various properties. Notably, γ‐GeSe is expected to exhibit low lattice thermal conductivity.^[^ ^46^, ^47^, ^64^ ^]^ The identified fully randomized stacking warrants further study of its effect on thermal conductivity and thermoelectric properties of γ‐GeSe. Moreover, numerous vdW crystals have been reported to undergo structural phase transition by interlayer sliding during temperature changes.^[^ ^62^, ^65^, ^66^, ^67^, ^68^, ^69^, ^70^ ^]^ Since the sliding energy barriers of other vdW crystals are comparable to that of γ‐GeSe, it may be necessary to account for the heat‐induced disordering effect, as unintentional stacking changes can pose significant challenges in analysis.

## 4 Experimental Section

### Sample Preparation and Characterizations

γ‐GeSe crystals were grown by chemical vapor deposition method using Au nanoparticle as catalysis.^[^ ^44^ ^]^ Cross‐sectional TEM samples were prepared by FIB (crossbeam 540, ZEISS). STEM image and SAED pattern were obtained using JEOL double Cs‐corrected ARM‐200F operated at 200 kV. The SHG measurements were performed using a home‐built microscope. A picosecond supercontinuum laser (NKT Photonics, SuperK Extreme) coupled with a wavelength selector (Photon, etc., LLTF Contrast) was used as the excitation source, with a 1064 nm wavelength chosen. A 50X confocal objective lens (NA = 0.7) was employed with 1 mW power. The SHG signal was collected in reflection geometry with appropriate short‐pass filters. The spectrometer (Princeton Instruments, IsoPlane320) with 300 grooves mm^−1^ grating was used, and the signal was detected with a back‐illuminated charge‐coupled device (Princeton Instruments, PIXIS 400BRX) detector. The SHG intensity was obtained by fitting the spectrum with a Gaussian function. The samples were raster‐scanned to generate SHG images.

### Device Fabrication and Measurements

γ‐GeSe was transferred to 300 nm SiO_2_/Si substrate by dry transfer method using Polydimethylsiloxane (PDMS). Electrode patterning was done by the standard e‐beam lithography (PIONEER Two, Raith GmbH), and the electrode metals (Au 100nm/Ti 10 nm or Au 100nm/Pt 10 nm) were deposited by DC sputtering. The electrical measurement was performed using a parameter analyzer (Keithley 4200A‐SCS) at 10^−6^ ∼ 10^−7^ torr.

### First‐Principles Calculations

The Vienna Ab initio Simulation Package (VASP) code was used to perform all DFT calculations employing the projector augmented wave (PAW) method.^[^ ^71^, ^72^, ^73^, ^74^ ^]^ Within the PAW approach, the valence electron configurations of Ge and Se were explicitly considered as 3d^10^ 4s^2^ 4p^2^ and 4s^2^ 4p^6^, respectively. The kinetic cutoff energy for the plane wave basis set was set to 500 eV, and the irreducible Brillouin zone integration was sampled using Γ‐centered k‐point meshes with a spacing of 0.15 Å^−1^. The non‐local vdW‐DF‐cx was used as the exchange‐correlation functional to describe the interlayer vdW interaction.^[^ ^75^, ^76^ ^]^

For modeling various stacking sequences of γ‐GeSe, the fully optimized AB' stacked γ‐GeSe with a force tolerance criterion of 0.01 eV Å^−1^ and energy tolerance of 10^−5^ eV was obtained. Then the optimized unit cell vectors and atomic positions was used to build other higher stacking sequence models, and relaxed the atoms and the lattice vectors in the superlattice only along the *z*‐direction, using the same convergence criteria. To calculate the interlayer sliding energies, the energy differences were obtained by displacing the upper layer Se atoms in AB′ stacked γ‐GeSe (see Figure 6b) along a uniform grid with increments of 0.418 and 0.434 Å along the cartesian x‐ and y‐coordinates, respectively. At each grid point, the Se atoms were fully relaxed in all directions, while the unit cell vectors and Ge atoms were relaxed only along the z‐direction.

### Finite Element Method

The Joule heating simulation was investigated using the AC/DC and heat transfer modules of COMSOL Multiphysics software. A steady‐state condition was employed to evaluate the heat transfer in the γ‐GeSe device. The simulation primarily considered the electrical and thermal conductivities of the materials and the interfaces.

## Conflict of Interest

The authors declare no conflict of interest.

## Supporting information



Supporting Information

## Data Availability

The data that support the findings of this study are available from the corresponding author upon reasonable request.

## References

[1] Z. Lin , Y. Huang , X. Duan , Nat. Electron. 2019, 2, 378.

[2] S. M. Akkanen , H. A. Fernandez , Z. Sun , Adv. Mater. 2022, 34, 2110152. 10.1002/adma.202110152 35139583

[3] D. Jiang , Z. Liu , Z. Xiao , Z. Qian , Y. Sun , Z. Zeng , R. Wang , J. Mater. Chem. A 2022, 10, 89.

[4] A. Giri , G. Park , U. Jeong , Chem. Rev. 2023, 123, 3329. 36719999 10.1021/acs.chemrev.2c00455 PMC10103142

[5] Z. Guan , H. Hu , X. Shen , P. Xiang , N. Zhong , J. Chu , C. Duan , Adv. Electron. Mater. 2020, 6, 1900818.

[6] S. Hong , C. Hong , S. Lee , M. Jang , C. Jang , Y. Lee , L. J. Widiapradja , S. Park , K. Kim , Y. Son , J. Yook , S. Im , Sci. Adv. 2023, 9, eadh9770. 37467332 10.1126/sciadv.adh9770 PMC10355828

[7] P. Ranjan , S. Gaur , H. Yadav , A. B. Urgunde , V. Singh , A. Patel , K. Vishwakarma , D. Kalirawana , R. K Gupta , Nano Converg. 2022, 9, 26. 35666392 10.1186/s40580-022-00317-7 PMC9170864

[8] I. Roh , S. H. Goh , Y. Meng , J. S. Kim , S. Han , Z. Xu , H. E. Lee , Y. Kim , S. H. Bae , Nano Converg. 2023, 10, 20. 37120780 10.1186/s40580-023-00369-3 PMC10149550

[9] H. W. Guo , Z. Hu , Z. B. Liu , J. G. Tian , Adv. Funct. Mater. 2020, 31, 2007810.

[10] H. Yoo , R. Engelke , S. Carr , S. Fang , K. Zhang , P. Cazeaux , S. H. Sung , R. Hovden , A. W. Tsen , T. Taniguchi , K. Watanabe , G. C. Yi , M. Kim , M. Luskin , E. B. Tadmor , E. Kaxiras , P. Kim , Nat. Mater. 2019, 18, 448. 30988451 10.1038/s41563-019-0346-z

[11] H. Bergeron , D. Lebedev , M. C. Hersam , Chem. Rev. 2021, 121, 2713. 33555868 10.1021/acs.chemrev.0c00933

[12] J. Srour , M. Badawi , F. El Haj Hassan , A. Postnikov , J. Chem. Phys. 2018, 149, 054106. 30089367 10.1063/1.5030539

[13] Y. Zhang , Y. Lee , W. Zhang , D. Song , K. Lee , Y. Zhao , H. Hao , Z. Zhao , S. Wang , K. Kim , N. Liu , Adv. Funct. Mater. 2023, 33, 2212210.

[14] K. Lee , J. Park , S. Choi , Y. Lee , S. Lee , J. Jung , J. Y. Lee , F. Ullah , Z. Tahir , Y. S. Kim , G. H. Lee , K. Kim , Nano Lett. 2022, 22, 4677. 35674452 10.1021/acs.nanolett.2c00550

[15] E. Sutter , H. P. Komsa , A. A. Puretzky , R. R. Unocic , P. Sutter , ACS Nano 2022, 16, 21199. 36413759 10.1021/acsnano.2c09172

[16] L. Li , M. Wu , ACS Nano 2017, 11, 6382. 28602074 10.1021/acsnano.7b02756

[17] S. Li , F. Wang , Y. Wang , J. Yang , X. Wang , X. Zhan , J. W. He , Adv. Mater. 2024, 36, 2301472. 10.1002/adma.202301472 37363893

[18] P. Meng , Y. Wu , R. Bian , E. Pan , B. Dong , X. Zhao , J. Chen , L. Wu , Y. Sun , Q. Fu , Q. Liu , D. Shi , Q. Zhang , Y. W. Zhang , Z. L. F. Liu , Nat. Commun. 2022, 13, 7696. 36509811 10.1038/s41467-022-35339-6 PMC9744910

[19] K. Ko , A. Yuk , R. Engelke , S. Carr , J. Kim , D. Park , H. Heo , H. M. Kim , S. G. Kim , H. Kim , T. Taniguchi , K. Watanabe , H. Park , E. Kaxiras , S. M. Yang , P. Kim , H. Yoo , Nat. Mater. 2023, 22, 992. 37365226 10.1038/s41563-023-01595-0

[20] M. Vizner Stern , Y. Waschitz , W. Cao , I. Nevo , K. Watanabe , T. Taniguchi , E. Sela , M. Urbakh , M. Hod , O. B. Shalom , Science 2021, 372, 1462. 10.1126/science.abe8177 34112727

[21] X. Wang , K. Yasuda , Y. Zhang , S. Liu , K. Watanabe , T. Taniguchi , J. Hone , L. J.‐H. Fu , Nat. Nanotechnol. 2022, 17, 367. 35039684 10.1038/s41565-021-01059-z

[22] F. Sui , M. Jin , Y. Zhang , R. Qi , Y. N. Wu , R. Huang , F. Yue , J. Chu , Nat. Commun. 2023, 14, 36. 36596789 10.1038/s41467-022-35490-0 PMC9810696

[23] Z. Fei , W. Zhao , T. A. Palomaki , B. Sun , M. K. Miller , Z. Zhao , J. Yan , X. Xu , D. H. Cobden , Nature 2018, 560, 336. 30038286 10.1038/s41586-018-0336-3

[24] Y. Wan , T. Hu , X. Mao , J. Fu , K. Yuan , Y. Song , X. Gan , X. Xu , M. Xue , X. Cheng , C. Huang , J. Yang , L. Dai , H. Zeng , E. Kan , Phys. Rev. Lett. 2022, 128, 067601. 35213175 10.1103/PhysRevLett.128.067601

[25] F. Sui , H. Li , R. Qi , M. Jin , Z. Lv , M. Wu , X. Liu , Y. Zheng , B. Liu , R. Ge , Y. N. Wu , R. Huang , F. Yue , J. Chu , C. Duan , Nat. Commun. 2024, 15, 3799. 38714769 10.1038/s41467-024-48218-z PMC11076638

[26] R. Bian , R. He , E. Pan , Z. Li , G. Cao , P. Meng , J. Chen , Q. Liu , Z. Zhong , W. Li , F. Liu , Science 2024, 385, 57. 38843352 10.1126/science.ado1744

[27] J. Xiao , Y. Wang , H. Wang , C. D. Pemmaraju , S. Wang , P. Muscher , E. J. Sie , Nyby , T. P. Devereaux , X. B Qian , Nat. Phys. 2020, 16, 1028.

[28] P. Sharma , F. Xiang , D. Shao , D. Zhang , E. Tsymbal , A. S. Hamilton , Sci. Adv. 2019, 5, eaax5080. 31281902 10.1126/sciadv.aax5080 PMC6611688

[29] J. Li , Y. Xie , C. Zhang , K. Ma , F. Liu , W. Ao , Y. Li , C. Zhang , ACS Appl. Mater. Interfaces 2019, 11, 20064. 31091077 10.1021/acsami.9b04984

[30] Y. Jiang , J. Dong , H. L. Zhuang , J. Yu , B. Su , H. Li , J. Pei , F. H. Sun , M. Zhou , H. Hu , J. W. Li , Z. Han , B. P. Zhang , T. Mori , J. F. Li , Nat. Commun. 2022, 13, 6087. 36241619 10.1038/s41467-022-33774-z PMC9568533

[31] E. Hadland , H. Jang , M. Falmbigl , R. Fischer , D. L. Medlin , D. G. Cahill , D. C. Johnson , Chem. Mater. 2019, 31, 5699.

[32] Y. Lu , Y. Zhou , W. Wang , M. Hu , X. Huang , D. Mao , S. Huang , L. Xie , P. Lin , B. Jiang , B. Zhu , J. Feng , J. Shi , Q. Lou , Y. Huang , J. Yang , J. Li , G. Li , J. He , Nat. Nanotechnol. 2023, 18, 1281. 37500776 10.1038/s41565-023-01457-5

[33] L. Xu , Y. Xiao , S. Wang , B. Cui , D. Wu , X. Ding , L. D. Zhao , Nat. Commun. 2022, 13, 6449. 36307447 10.1038/s41467-022-34227-3 PMC9616947

[34] S. Zheng , B. Zhang , Z. Zhou , A. Li , G. Han , X. Lu , G. Wang , X. Han , X. Zhou , Appl. Phys. Lett. 2024, 125, 051901.

[35] S. E. Kim , F. Mujid , A. Rai , F. Eriksson , J. Suh , P. Poddar , A. Ray , C. Park , E. Fransson , Y. Zhong , D. A. Muller , P. Erhart , D. G. P. Cahill , Nature 2021, 597, 660. 34588671 10.1038/s41586-021-03867-8 PMC8481126

[36] E. C. Hadland , H. Jang , N. Wolff , R. Fischer , A. C. Lygo , G. Mitchson , D. Li , L. Kienle , D. G. Cahill , D. C. Johnson , Nanotechnology 2019, 30, 285401. 30645979 10.1088/1361-6528/aafea2

[37] H. Li , W. Zheng , X. Liu , W. Liu , Q. Zhu , Physica E Low Dimens. Syst. Nanostruct. 2023, 148, 115658.

[38] E. Chavez‐Angel , P. Tsipas , P. Xiao , M. T. Ahmadi , A. H. S. Daaoub , H. Sadeghi , C. M. Sotomayor Torres , A. Dimoulas , A. E. Sachat , Nano Lett. 2023, 23, 6883. 37467035 10.1021/acs.nanolett.3c01280 PMC10416569

[39] G. Honjo , S. Miyake , T. Tomita , Acta Crystallogr. 1950, 3, 396.

[40] R. Ribeiro‐Palau , C. Zhang , K. Watanabe , T. Taniguchi , J. Hone , C. R. Dean , Science 2018, 361, 690.

[41] Z. Gao , T. R. Wei , T. Deng , P. Qiu , W. Xu , Y. Wang , L. Chen , X. Shi , Nat. Commun. 2022, 13, 7491. 36470897 10.1038/s41467-022-35229-x PMC9723169

[42] J. Tan , H. Zhang , X. Wang , Y. Wang , J. Wang , H. Zhang , E. Ma , W. Zhang , Mater. Today 2024, 10.1016/j.mattod.2024.08.029.

[43] J. Jang , J. Kim , D. Sung , J. H. Kim , J. E. Jung , S. Lee , J. Park , C. Lee , H. Bae , S. Im , K. Park , Y. J. Choi , S. Hong , K. Kim , Nano Lett. 2023, 23, 3144. 37026614 10.1021/acs.nanolett.2c04425

[44] S. Lee , J. E. Jung , H. G. Kim , Y. Lee , J. M. Park , J. Jang , S. Yoon , A. Ghosh , M. Kim , J. Kim , W. Na , J. Kim , H. J. Choi , H. Cheong , K. Kim , Nano Lett. 2021, 21, 4305. 33970636 10.1021/acs.nanolett.1c00714

[45] J. Park , Je , J. Kim , J. M. Park , J. E. Jung , H. Cheong , S. W. Lee , K. Kim , Nano Converg. 2024, 11, 29. 39009919 10.1186/s40580-024-00436-3 PMC11250563

[46] B. Wang , X. Yan , X. Cui , Y. Cai , ACS Appl. Nano Mater. 2022, 5, 15441.

[47] H. Minhas , S. Das , B. Pathak , ACS Appl. Energy Mater. 2022, 5, 9914.

[48] H. Kim , H. J. Choi , J. Mater. Chem. C 2021, 9, 9683.

[49] S. Shi , K. R. Hao , X. Y. Ma , Q. B. Yan , G. Su , J. Phys. Condens. Matter 2023, 35, 385501. 10.1088/1361-648X/acdd3e 37295439

[50] S. J. Magorrian , V. Zólyomi , N. D. Drummond , Phys. Rev. B 2021, 103, 094118.

[51] A. M. Mio , P. M. Konze , A. Meledin , M. Küpers , M. Pohlmann , M. Kaminski , R. Dronskowski , J. Mayer , M. Wuttig , Adv. Funct. Mater. 2019, 29, 1902332.

[52] M. Kupers , P. M. Konze , S. Maintz , S. Steinberg , A. M. Mio , O. Cojocaru‐Miredin , M. Zhu , M. Muller , M. Luysberg , J. Mayer , M. Wuttig , R. Dronskowski , Angew. Chem., Int. Ed. 2017, 56, 10204. 10.1002/anie.201612121 28194844

[53] J. Jung , S. Lee , H. Kang , M. Jang , J. Park , M. Petri , H. Lipsanen , Z. Sun , H. H. Yoon , K. Kim , J. Mater. Chem. C 2024, 12, 9662.

[54] J. L. Battaglia , A. Kusiak , V. Schick , A. Cappella , C. Wiemer , M. Longo , E. Varesi , J. Appl. Phys. 2010, 107, 044314.

[55] A. R. Khan , L. Zhang , K. Ishfaq , A. Ikram , T. Yildrim , B. Liu , S. Rahman , Y. Lu , Adv. Funct. Mater. 2021, 32, 2105259.

[56] Y. Li , Y. Rao , K. F. Mak , Y. You , S. Wang , C. R. Dean , T. F. Heinz , Nano Lett. 2013, 13, 3329. 23718906 10.1021/nl401561r

[57] J. Xiao , H. Zhu , Y. Wang , W. Feng , Y. Hu , A. Dasgupta , Y. Han , Y. Wang , D. A. Muller , L. W. Martin , P. Z. Hu , Phys. Rev. Lett. 2018, 120, 227601. 29906143 10.1103/PhysRevLett.120.227601

[58] Y. Yuan , P. Liu , H. Wu , H. Chen , W. Zheng , G. Peng , Z. Zhu , M. Zhu , J. Dai , S. Qin , K. S. Novoselov , ACS Nano 2023, 17, 17897. 37698446 10.1021/acsnano.3c03795

[59] A. M. Popov , I. V. Lebedeva , A. A. Knizhnik , Y. E. Lozovik , B. V. Potapkin , Phys. Rev. B 2011, 84, 045404.

[60] G. Constantinescu , A. Kuc , T. Heine , Phys. Rev. Lett. 2013, 111, 036104. 23909342 10.1103/PhysRevLett.111.036104

[61] H. Jiang , L. Li , Y. Wu , R. Duan , K. Yi , L. Wu , C. Zhu , L. Luo , M. Xu , L. Zheng , X. Gan , W. Zhao , X. Wang , Z. Liu , Adv. Mater. 2024, 36, 2400670. 10.1002/adma.202400670 38830613

[62] S. Cha , G. Lee , S. Lee , S. H. Ryu , Y. Sohn , G. An , C. Kang , M. Kim , K. Kim , A. Soon , K. S. Kim , Nat. Commun. 2023, 14, 1981. 37031234 10.1038/s41467-023-37740-1 PMC10082779

[63] N. Sivadas , S. Okamoto , X. Xu , C. J. Fennie , D. Xiao , Nano Lett. 2018, 18, 7658. 30408960 10.1021/acs.nanolett.8b03321

[64] Z. Shu , B. Wang , X. Cui , X. Yan , H. Yan , H. Jia , Y. Cai , Chem. Eng. J. 2023, 454, 140242.

[65] M. A. McGuire , H. Dixit , V. R. Cooper , B. C. Sales , Chem. Mater. 2015, 27, 612.

[66] J. Meseguer‐Sanchez , C. Popescu , J. L. Garcia‐Munoz , H. Luetkens , G. Taniashvili , E. Navarro‐Moratalla , Z. Guguchia , E. J. G. Santos , Nat. Commun. 2021, 12, 6265. 34725340 10.1038/s41467-021-26342-4 PMC8560937

[67] M. Jang , S. Lee , F. Cantos‐Prieto , I. Kosic , Y. Li , A. R. C. McCray , M. H. Jung , J. Y. Yoon , L. Boddapati , F. L. Deepak , H. Y. Jeong , C. M. Phatak , E. J. G. Santos , E. Navarro‐Moratalla , K. Kim , Nat. Commun. 2024, 15, 5925. 39009625 10.1038/s41467-024-50314-z PMC11251270

[68] F. T. Huang , L. S. Joon , S. Singh , J. Kim , L. Zhang , J. W. Kim , M. W. Chu , K. M. Rabe , D. Vanderbilt , S. W. Cheong , Nat. Commun. 2019, 10, 4211. 31527602 10.1038/s41467-019-11949-5 PMC6746811

[69] Y. Cheon , S. Y. Lim , K. Kim , H. Cheong , ACS Nano 2021, 15, 2962. 33480685 10.1021/acsnano.0c09162

[70] J. L. Hart , L. Bhatt , Y. Zhu , M. G. Han , E. Bianco , S. Li , D. J. Hynek , J. A. Schneeloch , Y. Tao , D. Louca , P. Guo , Y. Zhu , F. Jornada , E. J. Reed , L. F. Kourkoutis , J. J. Cha , Nat. Commun. 2023, 14, 4803. 37558697 10.1038/s41467-023-40528-y PMC10412583

[71] G. Kresse , J. Hafner , Phys. Rev. B 1993, 47, 558. 10.1103/physrevb.47.558 10004490

[72] G. Kresse , J. Furthmuller , Phys. Rev. B 1996, 54, 11169. 10.1103/physrevb.54.11169 9984901

[73] G. Kresse , D. Joubert , Phys. Rev. B 1999, 59, 1758.

[74] P. E. Blochl , Phys. Rev. B 1994, 50, 17953. 10.1103/physrevb.50.17953 9976227

[75] K. Berland , P. Hyldgaard , Phys. Rev. B 2014, 89, 035412.

[76] K. Berland , C. A. Arter , V. R. Cooper , K. Lee , B. I. Lundqvist , E. Schroder , T. Thonhauser , P. Hyldgaard , J. Chem. Phys. 2014, 140, 18A539. 10.1063/1.4871731 24832347

