# Supplementary material for: Thermally Induced Irreversible Disorder in Interlayer Stacking of γ‐GeSe
Source: Small. 2024 Oct 22;20(52):2407459. doi: 10.1002/smll.202407459 (PMC11673445; doi:10.1002/smll.202407459)
Supplement: Supplementary file 1 — Supporting Information [file SMLL-20-2407459-s001.docx]

**Supporting Information for**

**Thermally Induced Irreversible Disorder in Interlayer Stacking of γ-GeSe**

*Joonho Kim^1,†^, Giyeok Lee^2,†^, Sol Lee^1,3,†^, Jinsub Park^1^, Kihyun Lee^1,3^, Joong-Eon Jung^1^, Seungjae Lim^4^, Jeongsu Jang^1^, Heesun Bae^1^, Jae-Ung Lee^4^, Seongil Im^1^, Aloysius Soon^2,*^ and Kwanpyo Kim^1,3,*^*

^1^Department of Physics, Yonsei University, Seoul 03722, Korea.

^2^Department of Materials Science & Engineering, Yonsei University, Seoul 03722, Korea.

^3^Center for Nanomedicine, Institute for Basic Science (IBS), Seoul 03722, Korea.

^4^Department of Physics and Department of Energy Systems Research, Ajou University, Suwon 16499, Korea.

† Equal Contributions

*Address correspondence to A.S. (aloysius.soon@yonsei.ac.kr), K.K. (kpkim@yonsei.ac.kr)

Supporting Note 1. Finite element method (FEM) simulation

To investigate the temperature change and the electric field in the γ-GeSe device under the bias, we used the AC/DC and heat transfer modules of COMSOL Multiphysics software. The equations for the equilibrium conditions are expressed as follows:

$\mathbf{J}=\sigma\mathbf{E}=-\sigma(\boldsymbol{\nabla}V$) (1)

$Q_{e}=\mathbf{J}\boldsymbol{\cdot E}\boldsymbol{=}-\boldsymbol{\nabla\cdot(}\kappa\boldsymbol{\nabla}T)$ (2)

where J is the current density, σ is the electrical conductivity, E is the electric field, V is the electric potential, Q_e_ is the resistive heating due to the electric current, and κ is the thermal conductivity. As mentioned in the main text, we applied isotropic values for the electrical and thermal conductivity of γ-GeSe that are referenced from our previous works. ^[1,2]^ However, these values are experimentally known only for the in-plane (IP) direction, while the out-of-plane (OOP) value is unknown and cannot be ignored. Therefore, we examined the maximum temperature of the sample while varying the range of OOP/IP conductivity ratios from 0.1 to 1 **(Figure S4a, b)**. Generally, in IV-VI and V_2_-VI_3_ van der Waals materials, the OOP conductivities are lower than the IP conductivities, and the difference is less than a factor of 10, making this range setting reasonable. ^[3-6]^ As shown in the **Figure S4a and b**, the anisotropic effect on electrical conductivity is negligible while the anisotropic effect on thermal conductivity shows a difference of ~5% on the maximum temperature. Therefore, the impact of OOP conductivity is not significant in this study.

The electrical contact resistance and thermal boundary conductance (TBC) are also highly important. We compared two-terminal measurement and four-point probe measurement for each device to find the precise value of the contact resistance. We adopted TBC between the γ-GeSe and SiO_2_ of 16.7 MWm^-2^K^-1^ which is the value between the GST and SiO_2_. ^[7]^ Typically, TBC value range from several tens to several hundred MWm^-2^K^-1^, and within this range, the maximum temperature does not show significant variation **(Figure S4c)**. ^[8,9]^ Considering all these factors, although the exact values of OOP conductivities and TBC are not known, the simulation results presented in the main text are reasonable, and the possible errors do not significantly affect our conclusions.

Supporting Note 2. Multicollinearity analysis in DFT–parameterized model

To construct the DFT–parameterized model, we used 4 local geometric features from the nearest neighboring monolayer (denoted as $k$; **Figure S8b**) and 7 local geometric features from the next-nearest neighboring monolayer (denoted as $l$; **Figure S8c**) to formulate the linear equation.

The 4 local geometric features from the nearest neighboring monolayers are as follows: (i) The number of neighboring Se atoms on different hexagonal sublattice sites (labeled as $k_{\mathrm{NX}}$); (ii) the number of next-nearest Se atoms on the same hexagonal sublattice site as a Ge atom (denoted as $k_{\mathrm{NNX}}$); (iii) the number of Ge-Ge dimers in the neighboring monolayers that share the same hexagonal sublattice sites based on the next-nearest neighboring Ge atoms (denoted as $k_{\mathrm{NNM}}$); and (iv) the number of quadruple atomic units of neighboring monolayers that have different hexagonal sublattice configurations (i.e., clockwise ↔ counter-clockwise; labeled as $k_{\mathrm{flip}}$).

The 7 local geometric features from the next-nearest neighboring monolayer were first categorized into Type 1 to Type 3 based on the arrangement of Se and Ge atoms in the nearest neighboring monolayer, as shown in **Figure S8b**. We then tracked how these relate to each other in the next-nearest neighboring monolayer. The cases where the types are the same were labeled as $l_{11}$, $l_{22}$, and $l_{33}$, while the cases where the types are different were categorized into four terms, $l_{{11}_{\mathrm{flip}}}$, $l_{12}$, $l_{13}$, and $l_{23}$.

To minimize multicollinearity caused by high correlation among the above parameters, we constructed all possible linear equations using combinations ranging from 2 to 11 parameters, resulting in a total of 2,035 combinations ($\Sigma_{i=2}^{11}$*_n_C_r_*). For each linear equation, we calculated the variance inflation factor (VIF) as follows:

$\mathrm{VIF}_{i}=\frac{1}{1-R_{i}^{2}}$ , (3)

where $R_{i}^{2}$ is the $R^{2}$ value obtained by regressing the linear equation against all the other parameters. Here, we selected the combination with the highest $R^{2}$ value among those where the VIF values of all parameters were less than 3. This resulted in a linear equation consisting of 8 parameters, excluding $k_{\mathrm{NNX}}$, $l_{11}$, and $l_{33}$. By conducting first-principles calculations for all distinctive stacking sequences up to 4 monolayers and fitting the energy to the linear equation, the coefficients of $k_{\mathrm{NX}}$, $k_{\mathrm{NNM}}$, $k_{\mathrm{flip}}$, $l_{{11}_{\mathrm{flip}}}$, $l_{12}$, $l_{13}$, $l_{23}$, and $l_{22}$ were evaluated as –79.82, +4.62, –1.70, +0.36, +2.66, +0.46, +2.77, and +4.82 meV, respectively. The corresponding VIF values were measured as 1.695, 1.557, 1.933, 1.752, 1.776, 1.995, 1.585, and 1.411. For training (1 to 4 ML) and validation (5 to 6 ML), the $R^{2}$ value of were 0.99989 and 0.99985, respectively, with the RMSE value of 0.215 and 0.221 meV/cell/layer.


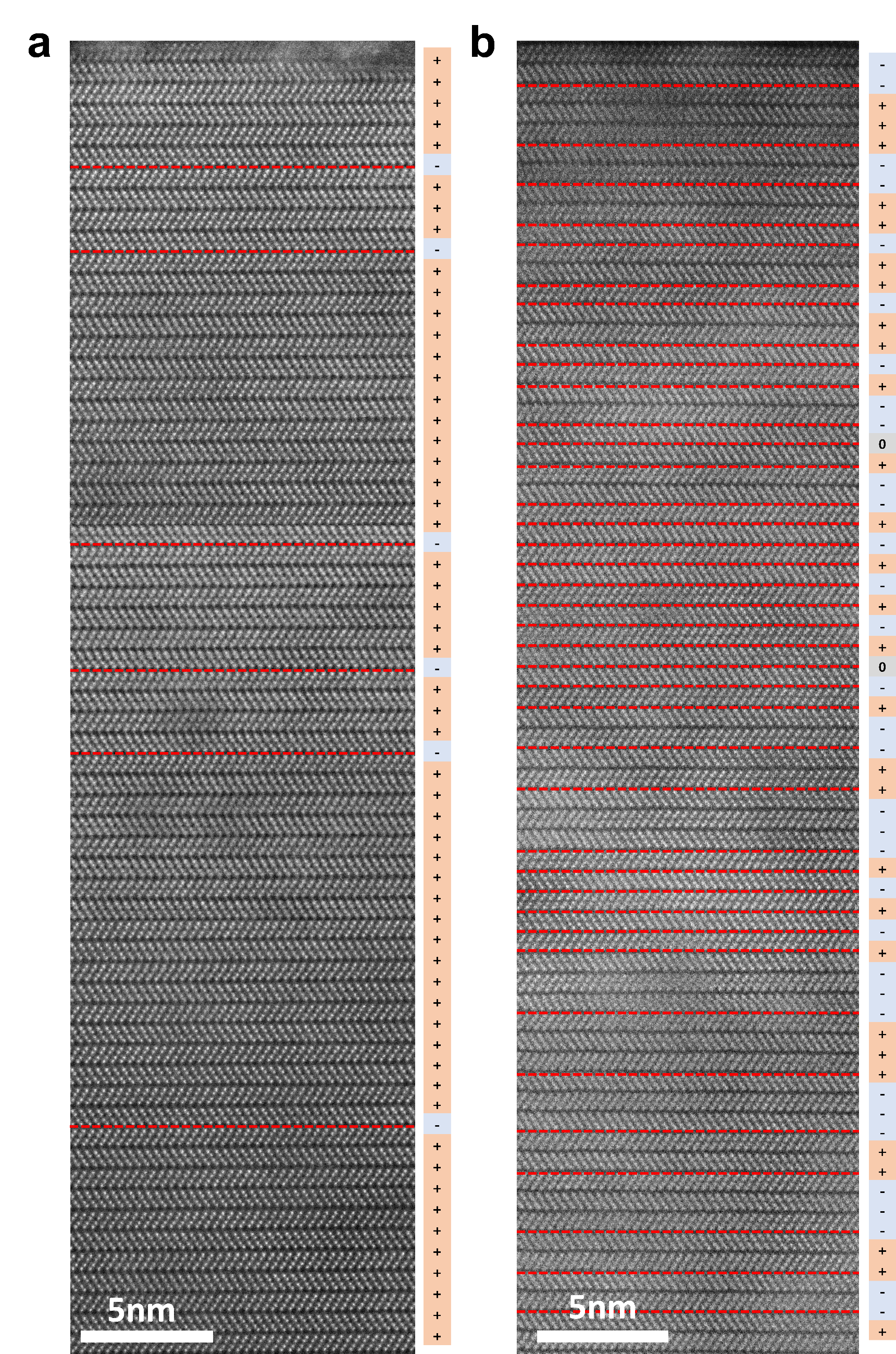


**Figure S1. Exemplary STEM images of γ-GeSe for stacking sequence investigation.** (a) STEM image of as-synthesized γ-GeSe along the [$10\bar{1}0$] zone axis. (b) STEM image of electrically perturbed γ-GeSe along the [$10\bar{1}0$] zone axis. Stacking faults are marked with the red dashed line.


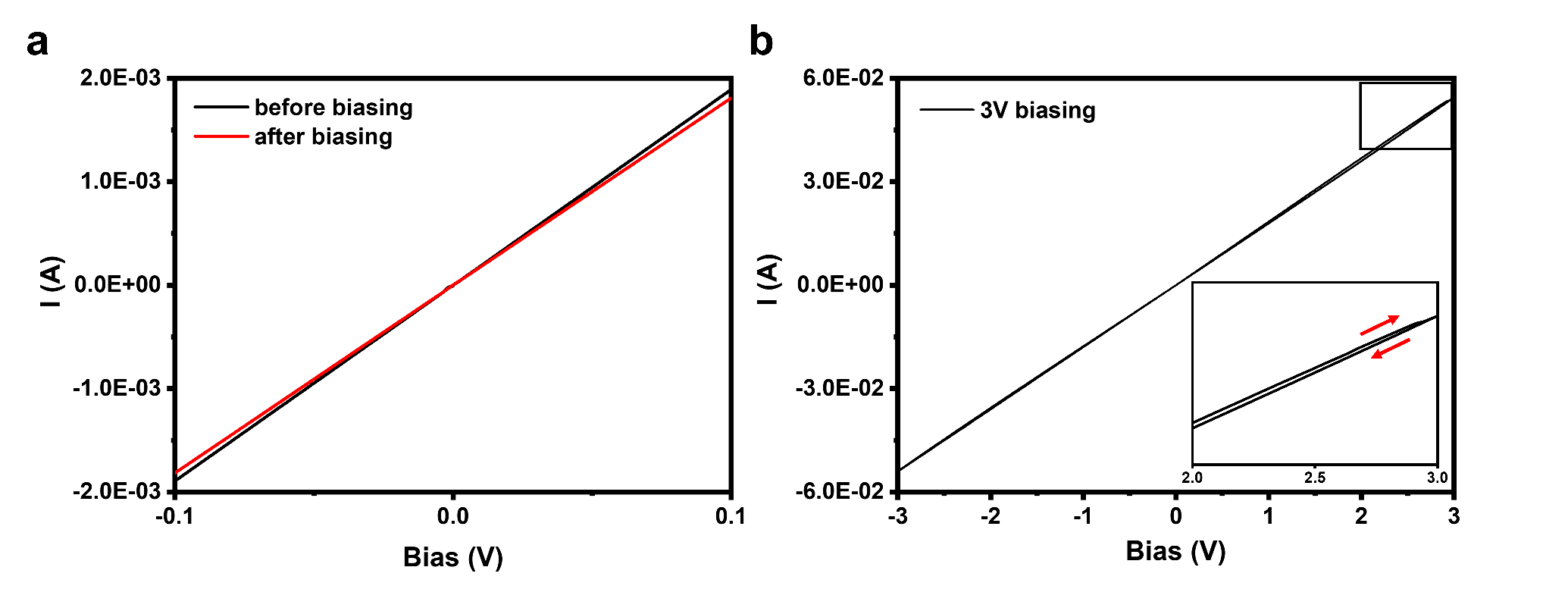


**Figure S2. Exemplary electrical measurement data from two-terminal γ-GeSe device.** (a) I-V characteristics of γ-GeSe at relatively low bias voltage. (b) I-V characteristics of γ-GeSe when applying relatively high bias voltage. The permanent resistance increase was observed.


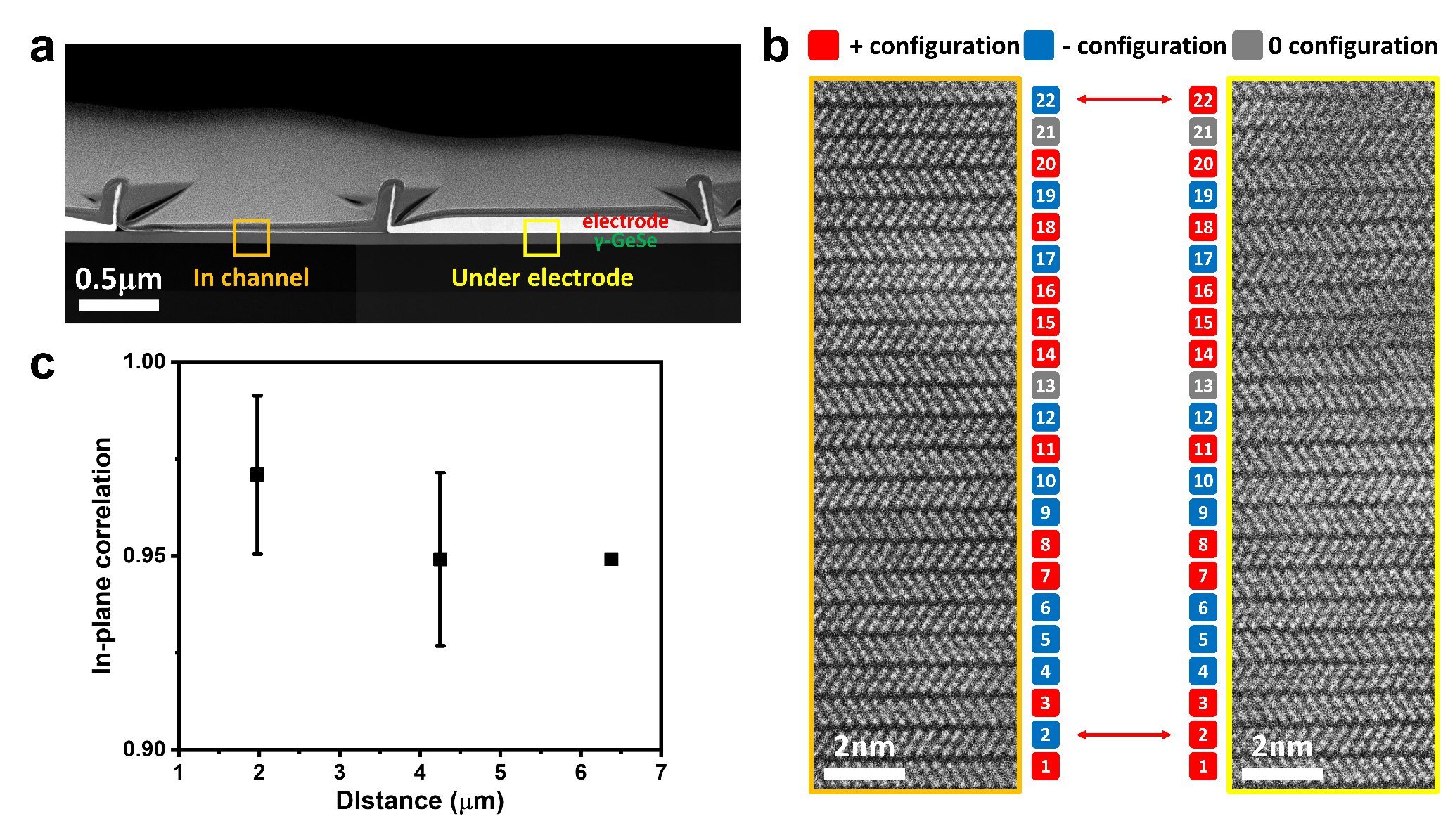


**Figure S3. In-plane stacking correlation of γ-GeSe after electrical perturbation.** (a) Low-magnification cross-sectional STEM image of the device. (b) Exemplary STEM images from the regions of orange and yellow boxes in panel a. The comparison of stacking sequences from two regions can be used to identify the discontinuity of layer shift in the in-plane direction. (c) Stacking sequence correlations as a function of distance.
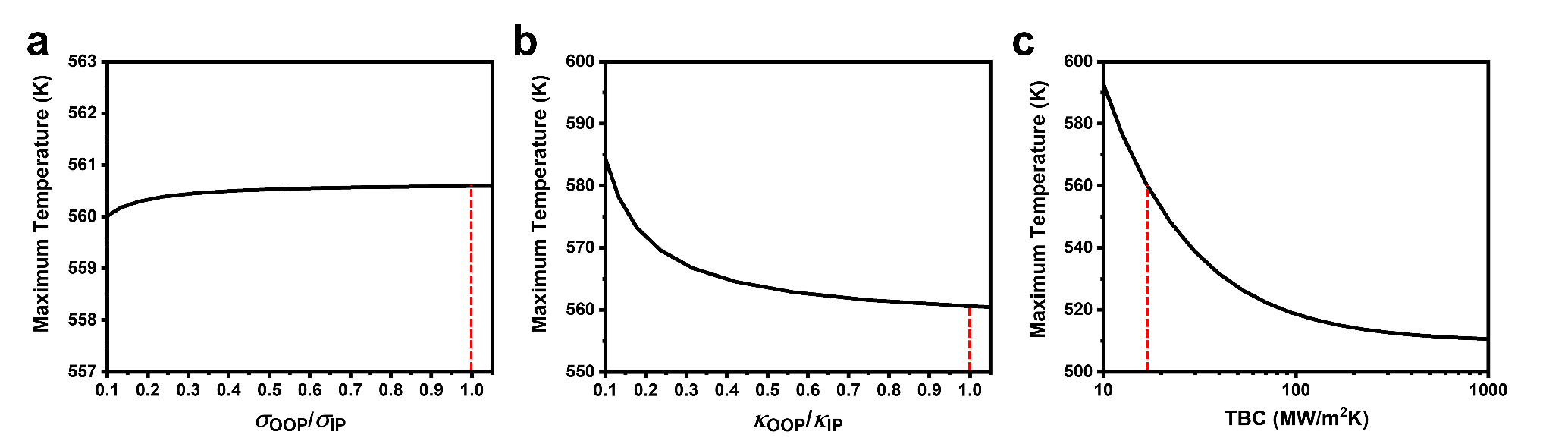


**Figure S4. FEM simulation on the effect of OOP conductivities and TBC.** (a) Simulated maximum temperature of γ-GeSe as a function of σ_OOP_/σ_IP_ ratio. (b) Simulated maximum temperature of γ-GeSe as a function of κ_OOP_/κ_IP_ ratio. (c) Simulated maximum temperature of γ-GeSe as a function of TBC value. Red dashed lines are the values used in the main text.


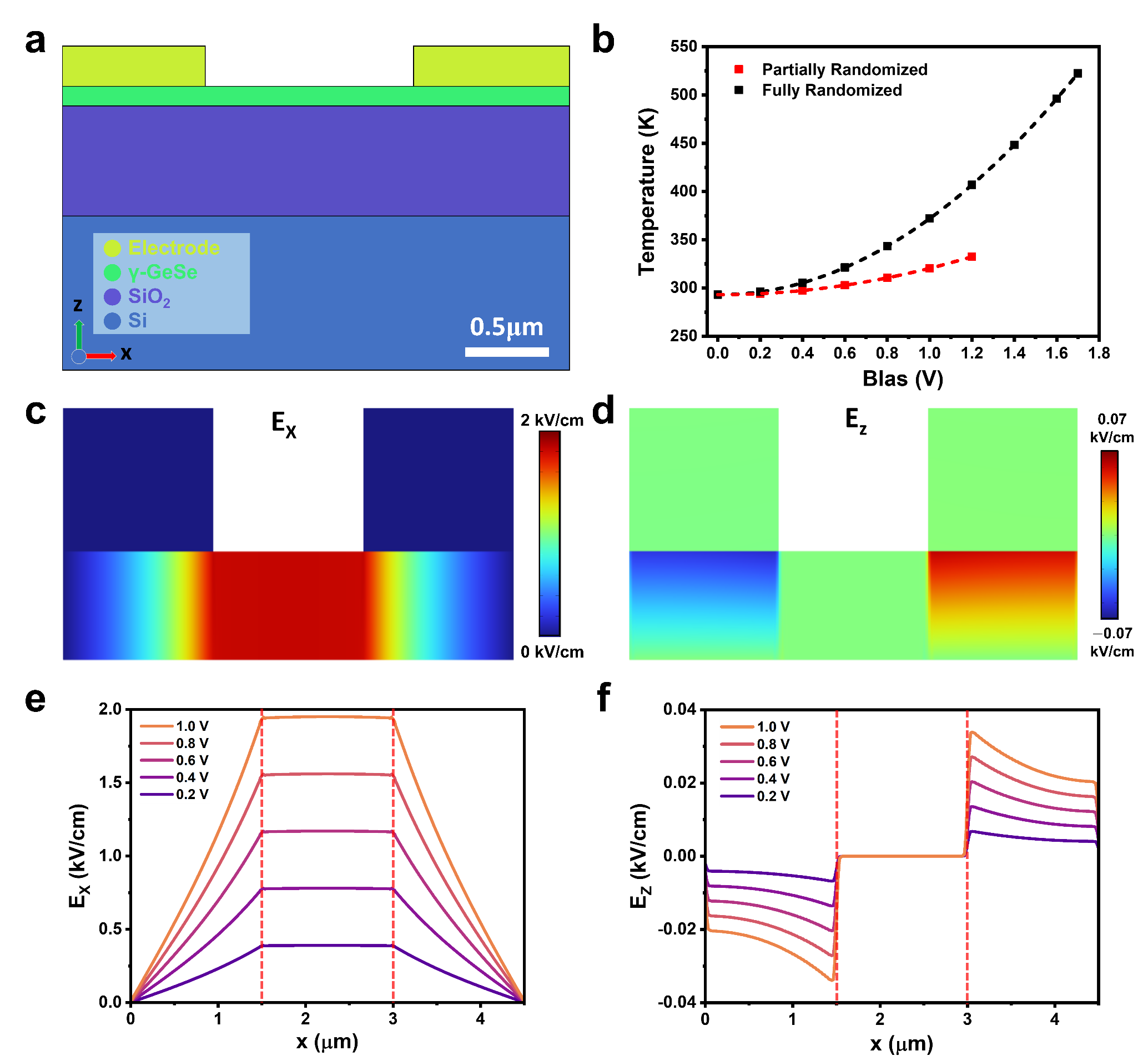


**Figure S5. FEM simulation of the γ-GeSe device.** (a) Side-view schematic of a γ-GeSe device for simulation. (b) Local maximum temperature of partially and fully randomized γ-GeSe under electrical biasing. (c) Horizontal component of the electric field. (d) Vertical component of the electric field. (e) Line profile of the horizontal component of the electrical field. (f) Line profile of the vertical component of the electrical field.


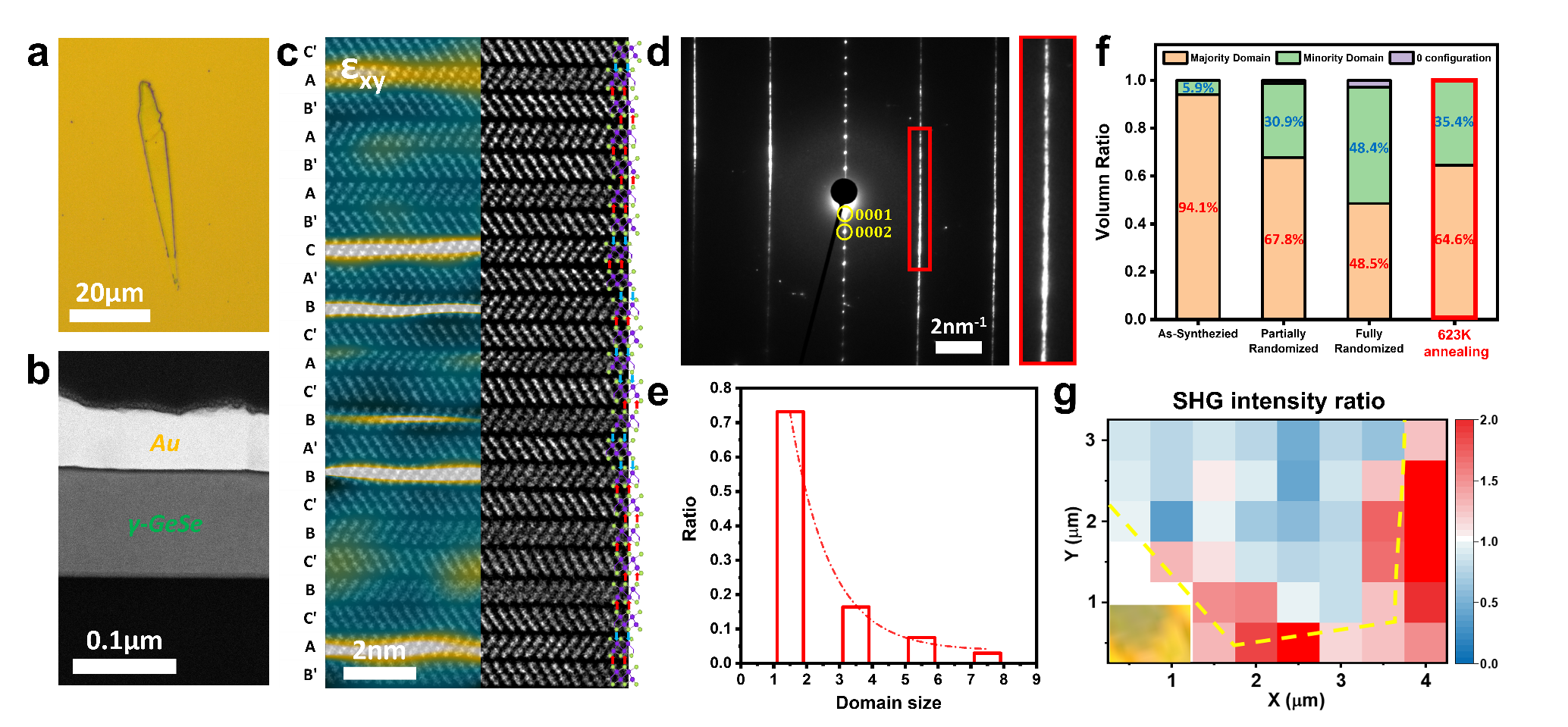


**Figure S6. Disordered stacking sequence in γ-GeSe by thermal annealing.** (a) γ-GeSe crystal after Au deposition and thermal annealing (b) cross-sectional TEM image of γ-GeSe shown in panel a. (c) Cross-sectional STEM image of γ-GeSe along the [$10\bar{1}0$] zone axis after thermal annealing. The overlaid strain εxy color map indicates the formation of significant number of SFs. (d) SAED pattern of annealed γ-GeSe along the [$10\bar{1}0$] zone axis. (e) Vertical domain size distributions after thermal annealing. The dotted curve is the fitting to data with an exponential decaying function. Total 131 layers from thermally treated sample are investigated. (f) Comparison of stacking domain ratio of different disordering methods. (g) SHG mapping of the intensity ratio between the pristine sample and the identical sample after thermal annealing. Yellow dashed line indicates the edge of the sample.


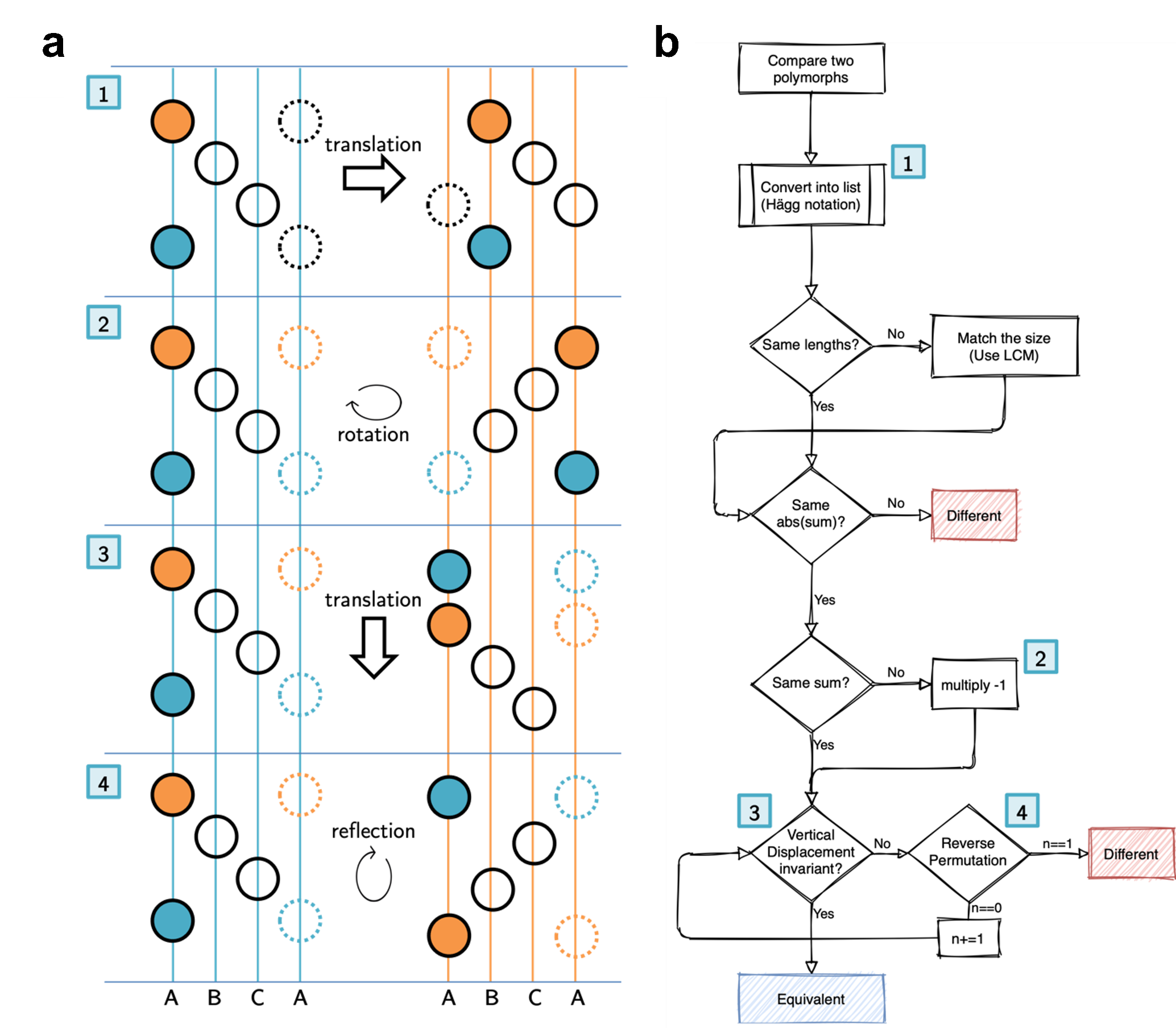


**Figure S7.** **Generation of unique stacking configurations in multilayer γ-GeSe.** (a) Schematic illustrations of four rigid structural operations of γ-GeSe. Se atoms are labelled as orange and turquoise circles (dashed hollow circles for repeated sites due to periodicity), and Ge atoms are labelled as black hollow circles. (b) Schematic illustrations of stacking sequence redundancy check algorithm.


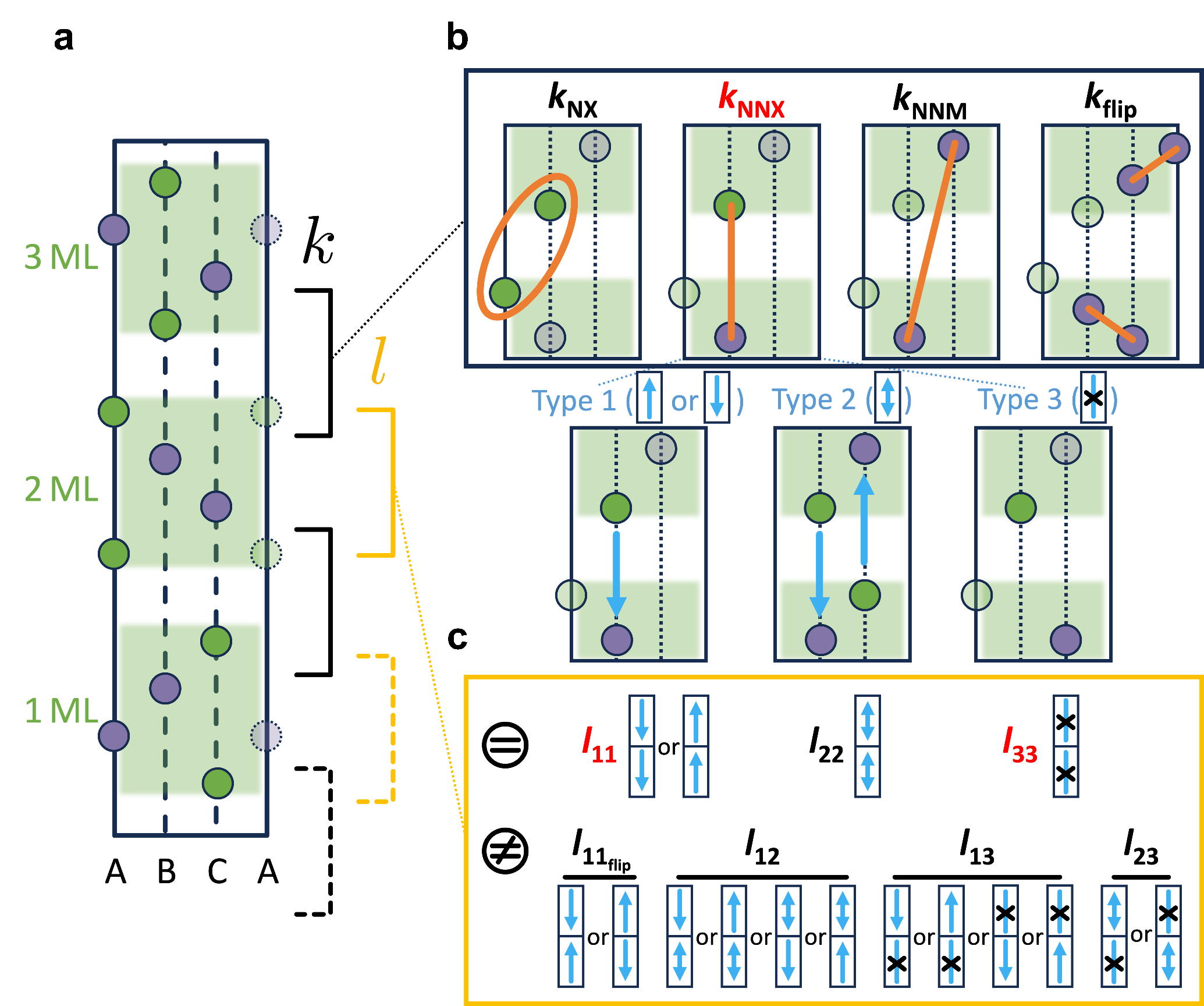


**Figure S8**. **Schematic illustration of 11 geometric features.** (a) 3 ML γ-GeSe model, and each monolayer is compartmentalized in green shades. Se and Ge atoms are labelled as green circles and purple circles, respectively. (b) 4 Geometric features, *k*, from the nearest neighboring monolayer. Specifically, for *k*_NNX_, there are 3 distinct ways to denote the geometric features, namely Type 1, Type 2, and Type 3. (c) Based on the different combinations of Types 1, 2, and 3, 16 geometric features may be obtained from the next-nearest neighboring monolayer which can be classified by 7 unique labels, *l*. Three features that colored in red are excluded in the linear fit based on variance inflation factor (VIF).


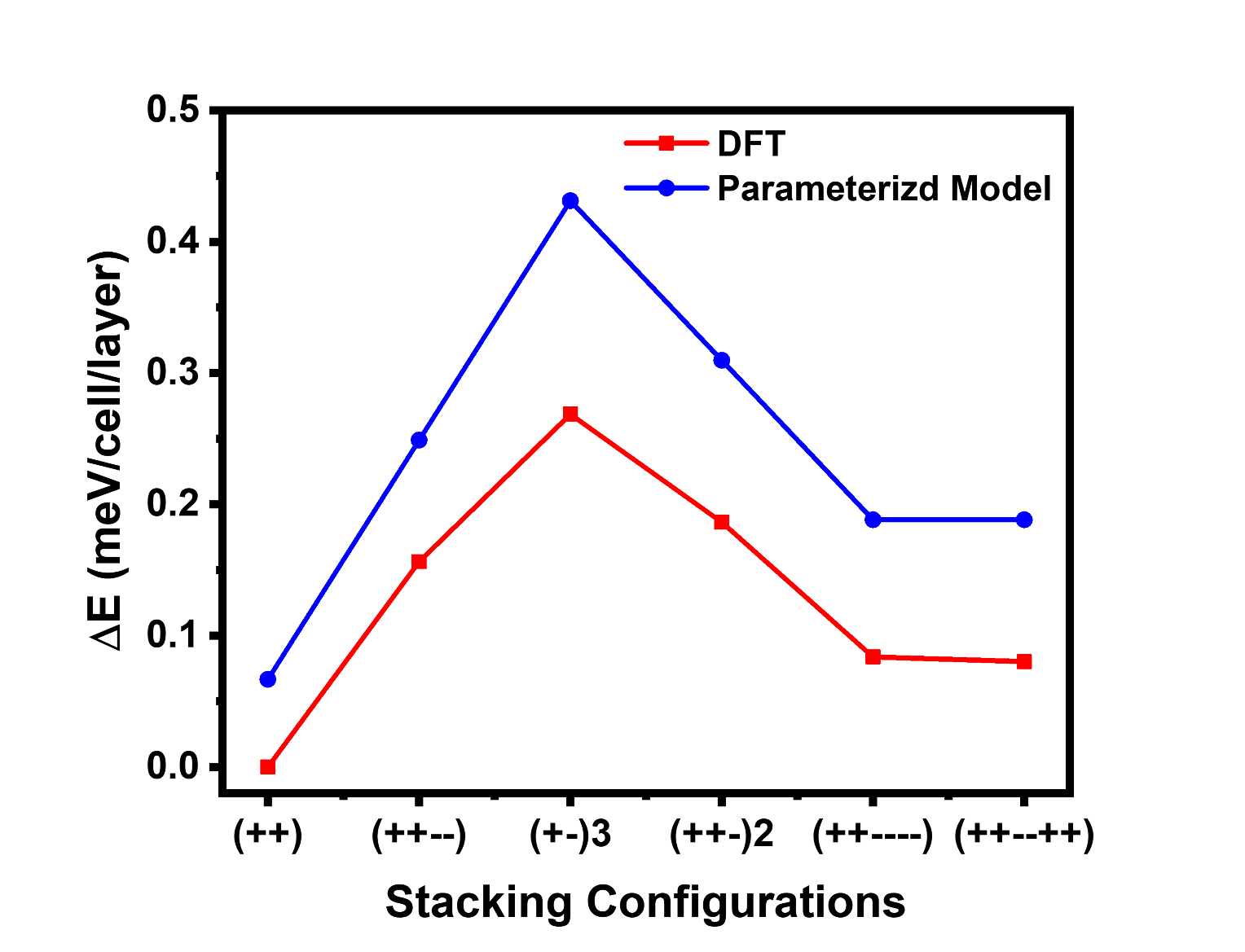


**Figure S9. Energy difference between stable stacking configurations.** Energy difference from stable stacking configurations from ordered stacking to randomized stacking. The model well predicts the energy correction from the next nearest monolayer.

| **N^th^ ML** | **# of structures at N^th^ ML** | **# of non-overlapping structures** |
| --- | --- | --- |
| 1 | 2 | 1 |
| 2 | 12 | 3 |
| 3 | 72 | 13 |
| 4 | 432 | 55 |
| 5 | 2,592 | 259 |
| 6 | 15,552 | 1,310 |

**Table S1.** **Generated γ-GeSe stacking configurations without and with consideration of overlapping structures.**

| **Material** | **Sliding energy barrier (meV atom^-1^)** | **Reference** |
| --- | --- | --- |
| Graphene | 2.1 | 59 |
| h-BN | 3.4 | 60 |
| MoS_2_ | 2.58 | 61 |
| PbI_2_ | 10.6 | 62 |
| CrI_3_ | 2.2 | 63 |
| **γ-GeSe** | **4.99** | **This work** |

**Table S2. Calculated sliding energy barriers of representative vdW materials.**

**References**

[1] Jang, J.; Kim, J.; Sung, D.; Kim, J. H.; Jung, J. E.; Lee, S.; Park, J.; Lee, C.; Bae, H.; Im, S.; Park, K.; Choi, Y. J.; Hong, S. Kim, K. Electrical Transport Properties Driven by Unique Bonding Configuration in γ-GeSe. *Nano Lett.* **2023,** *23*, 3144-3151.

[2] Park, J.; Je, Y.; Kim, J.; Park, J. M.; Jung, J. E.; Cheong, H.; Lee, S. W. Kim, K. Unveiling the Distinctive Mechanical and Thermal Properties of γ-GeSe. *Nano Converg.* **2024,** *11*, 29.

[3] Zhao, L. D.; Lo, S. H.; Zhang, Y.; Sun, H.; Tan, G.; Uher, C.; Wolverton, C.; Dravid, V. P. Kanatzidis, M. G. Ultralow Thermal Conductivity and High Thermoelectric Figure of Merit in SnSe Crystals. *Nature* **2014,** *508*, 373-377.

[4] Zhan, S.; Hong, T.; Qin, B.; Zhu, Y.; Feng, X.; Su, L.; Shi, H.; Liang, H.; Zhang, Q.; Gao, X.; Ge, Z. H.; Zheng, L.; Wang, D. Zhao, L. D. Realizing High-Ranged Thermoelectric Performance in PbSnS_2_ Crystals. *Nat. Commun.* **2022,** *13*, 5937.

[5] Kimberly, T. Q.; Ciesielski, K. M.; Qi, X.; Toberer, E. S. Kauzlarich, S. M. High Thermoelectric Performance in 2D Sb_2_Te_3_ and Bi_2_Te_3_ Nanoplate Composites Enabled by Energy Carrier Filtering and Low Thermal Conductivity. *ACS Appl. Electron. Mater.* **2024,** *6*, 2816-2825.

[6] Shi, Q.; Zhao, X.; Chen, Y.; Lin, L.; Ren, D.; Liu, B.; Zhou, C. Ang, R. Cu_2_Te Incorporation-Induced High Average Thermoelectric Performance in p-Type Bi_2_Te_3_ Alloys. *ACS Appl. Mater. Interfaces* **2022,** *14*, 45582-45589.

[7] Battaglia, J. L.; Kusiak, A.; Schick, V.; Cappella, A.; Wiemer, C.; Longo, M. Varesi, E. Thermal Characterization of the SiO_2_-Ge_2_Sb_2_Te_5_ Interface from Room Temperature up to 400°C. *J. Appl. Phys.* **2010,** *107*, 044314.

[8] Feng, T.; Zhou, H.; Cheng, Z.; Larkin, L. S. Neupane, M. R. A Critical Review of Thermal Boundary Conductance across Wide and Ultrawide Bandgap Semiconductor Interfaces. *ACS Appl. Mater. Interfaces* **2023,** *15*, 29655-29673.

[9] Giri, A. Hopkins, P. E. A Review of Experimental and Computational Advances in Thermal Boundary Conductance and Nanoscale Thermal Transport across Solid Interfaces. *Adv. Funct. Mater.* **2019,** *30*, 1903857.
